# Machine learning and deep learning for diagnosis of Polyendocrine Metabolic Ovarian Syndrome: systematic review and meta-analysis

**DOI:** 10.3389/fendo.2026.1889375

**Published:** 2026-07-30

**Authors:** Huaying Fan, Sifan Chen, Feiyan Cai, Chenjian Tang, Xiaohua Chen, Jiao Chen, Ping Wu

**Affiliations:** 1 Hospital of Chengdu University of Traditional Chinese Medicine, Chengdu, China; 2 Chengdu University of Traditional Chinese Medicine, Chengdu, China; 3 West China Hospital of Sichuan University, Chengdu, China

**Keywords:** artificial intelligence, deep learning, diagnostic accuracy, machine learning, meta-analysis, Polyendocrine Metabolic Ovarian Syndrome

## Abstract

**Background:**

Polyendocrine Metabolic Ovarian Syndrome (PMOS) is a prevalent endocrine disorder with a challenging, heterogeneous diagnosis. Machine learning (ML) and deep learning (DL) models show promise for automated diagnosis, but a quantitative synthesis of their accuracy is lacking.

**Objective:**

To evaluate the diagnostic accuracy of ML/DL models for PMOS and identify factors influencing performance.

**Methods:**

We searched MEDLINE, Web of Science, Embase, and Cochrane Library from inception to January 26, 2026. Studies developing or validating ML/DL models for PMOS diagnosis were included. Risk of bias was assessed using QUADAS-2. A random-effects model with the Hartung-Knapp-Sidik-Jonkman method was used to pool estimates. Hierarchical summary receiver operating characteristic curves were constructed. Heterogeneity was quantified (*I*^2^), and meta-regression and subgroup analyses explored sources of heterogeneity.

**Results:**

Fifty-six studies (60 datasets) were included. Pooled sensitivity was 0.92 (95% CI: 0.89-0.94; *I^2^* = 93.6%) and specificity 0.94 (95% CI: 0.91-0.96; I*^2^* = 93.4%), with an HSROC area under the curve of 0.99 (95% CI: 0.97-0.99). Prediction intervals were wide (sensitivity: 50%-99%; specificity: 53%-100%). Egger’s test suggested small-study effects (*P* < 0.05). Meta-regression identified data source (database vs. single-center, coefficient=1.91, *P* = 0.002) and modeling variables (ultrasound image vs. clinical parameters, coefficient=2.43, *P* = 0.007) as independent predictors of higher accuracy; model type was not significant after adjustment. Ultrasound image-based models achieved the highest accuracy (sensitivity 0.99, specificity 0.98). Notably, 32 studies did not report diagnostic criteria, and only 4 performed external validation.

**Conclusion:**

ML/DL models, particularly those using ultrasound imaging, demonstrate promising but conditional diagnostic accuracy for PMOS. However, poor reporting of diagnostic criteria, lack of external validation, and substantial heterogeneity limit current evidence. Future research must prioritize rigorous validation and adherence to reporting standards. While not yet ready for independent clinical use, these models hold promise as assistive tools to standardize ovarian assessment.

**Systematic review registration:**

https://www.crd.york.ac.uk/PROSPERO/view/CRD420251137107, identifier CRD420251137107.

## 1 Introduction

Polyendocrine metabolic ovarian syndrome (PMOS), formerly known as polycystic ovary syndrome (PCOS), represents the same clinical entity-characterized by hyperandrogenism, ovulatory dysfunction, and/or polycystic ovarian morphology-with the updated nomenclature reflecting an expanded understanding of its systemic metabolic nature rather than a distinct pathological condition (1). And this condition is affecting approximately 10-13% of women globally (2), with a prevalence rate as high as 10.01% in China (3). The clinical presentation of PMOS is highly heterogeneous, primarily characterized by menstrual irregularities, hyperandrogenism, and polycystic ovarian morphology. This condition is not only strongly associated with adverse reproductive outcomes such as infertility and pregnancy complications (including gestational diabetes and preeclampsia) (4–6) but also is frequently complicated by comorbidities including obesity, insulin resistance, type 2 diabetes, cardiovascular diseases, and psychological disorders (7–9), posing significant threats to patients’ long-term quality of life. Early and accurate diagnosis of PMOS is crucial for implementing effective interventions and preventing potential complications (10).

However, due to regional population differences and the complexity of the disease, the diagnosis and management of PMOS remain persistent challenges in the fields of gynecology and endocrinology (11). According to a World Health Organization (WHO) report, up to 70% of PMOS cases worldwide remain undiagnosed (12), and 30% of women experience a diagnostic delay of more than two years (13). Although the diagnostic and treatment criteria for PMOS were revised multiple times between 1990 and 2023 (10, 14–16), the current diagnostic process still faces several challenges. First, diagnosis relies on ultrasonography and detailed clinical and biochemical analyses-procedures that are time-consuming, labor-intensive, and require professional interpretation. Second, the diversity of clinical manifestations and the subjectivity of assessment may lead to diagnostic inconsistencies. Third, there is a lack of evaluation tools capable of integrating multidimensional information, making it difficult to achieve a comprehensive and precise description of the disease (17). Therefore, how to achieve early identification and diagnosis of PMOS has become an important research focus in the field.

In recent years, artificial intelligence (AI) technologies, particularly machine learning (ML) and deep learning (DL), have demonstrated significant potential in medical image analysis, disease prediction, and diagnostic assistance. AI models are capable of efficiently processing large-scale, multi-source complex data, automatically learning underlying patterns within them, thereby providing objective, reproducible, and precise diagnostic recommendations. This offers potential support in overcoming the limitations of manual diagnosis. ML and DL technologies have now been widely applied in the auxiliary diagnosis of various diseases, including lumbar spinal stenosis (18), peripheral artery disease (19), bipolar disorder (20), chronic obstructive pulmonary disease (21), Alzheimer’s disease (22), etc. In the context of PMOS, AI applications primarily encompass prediction, diagnosis, classification, and screening for complications, showing promising prospects. Numerous studies have confirmed that in PMOS-related diagnostic tasks, ML or DL models not only exhibit strong diagnostic performance but have even matched or surpassed the level of clinical experts (23). For instance, Zhao et al. developed a DL-based model for the automated recognition of polycystic ovary ultrasound images. The results showed that the model achieved area under the curve (AUC) values of 0.953, 0.973, and 0.967 in the training set, internal validation set, and external validation set, respectively (24). Moreover, the model evaluated a single ovary 50 times faster than clinicians, underscoring the significant value of AI tools in the diagnosis of PMOS (24).

Existing reviews have summarized the applications of ML and DL in various aspects of PMOS, such as diagnosis, risk prediction, classification, and elucidation of pathogenesis (17, 23). These reviews indicate that AI-based diagnostic tools can leverage large-scale medical records and imaging data to develop high-accuracy detection algorithms. Such algorithms can analyze patterns within the data and identify subtle signs and markers of PMOS that may be overlooked by clinicians. AI-based PMOS detection tools contribute to the digitization and automation of diagnostic processes, promoting earlier and more accurate diagnosis, thereby enabling timely intervention and improved patient outcomes (17). It is noteworthy that these existing reviews still lack a comprehensive integration and quantitative analysis of existing evidence to clarify the overall efficacy of AI models in PMOS diagnosis and its influencing factors. Therefore, this study is designed to thoroughly evaluate the accuracy of ML and DL models in diagnosing PMOS through systematic review and meta-analysis, with the aim of providing an evidence-based foundation for optimizing AI-assisted diagnostic strategies and facilitating their clinical translation in the future.

## 2 Materials and methods

### 2.1 Registration

This study was conducted in accordance with the Preferred Reporting Items for Systematic Reviews and Meta-Analyses (PRISMA) guidelines (25), the PRISMA 2020 expanded checklist, and the PRISMA extension for diagnostic test accuracy (DTA). It was prospectively registered on the International Prospective Register of Systematic Reviews (PROSPERO) platform with a registration number of CRD420251137107 (https://www.crd.york.ac.uk/PROSPERO/view/CRD420251137107). This systematic review followed the PROSPERO protocol with the following amendments. First, we expanded the search sources to include clinical trial registries and gray literature, and performed manual reference screening and citation tracking, which were not explicitly detailed in the original protocol. Second, the updated search strategy was peer-reviewed using the PRESS checklist, adding a quality control step. Third, to address the high heterogeneity encountered, we introduced additional statistical analyses not pre-specified: calculation of 95% prediction intervals, meta-regression to explore sources of heterogeneity, and Baujat plot to identify influential outliers. Fourth, small study effect was assessed using Egger’s linear regression test. All protocol deviations were documented, discussed, and approved by all review team members before implementation, and were undertaken to enhance the validity and interpretability of our findings.

### 2.2 Search strategy

We searched four databases, including MEDLINE via PubMed (NCBI), Web of Science Core Collection via Clarivate Analytics (Web of Science), Embase via Embase.com (Elsevier), and the Cochrane Library via Wiley Online Library. In addition, we searched clinical trial registries, namely ClinicalTrials.gov and the International Clinical Trials Registry Platform, as well as gray literature, primarily ACS Publications. We also manually screened the reference lists of included studies to identify relevant research and used the citation alert function in Web of Science to conduct ongoing citation tracking for studies included in the Web of Science Core Collection. For ongoing or unpublished studies, as well as cases where publications, conference proceedings, or clinical trial registrations did not provide complete information, we contacted the corresponding authors to obtain the relevant details. Finally, a PubMed related articles search was performed on all included articles to further identify relevant literature.

To enhance the transparency of the search and reporting processes, we followed the Preferred Reporting Items for Systematic Reviews and Meta-Analyses literature search extension (PRISMA-S) checklist (26). The search strategy combined Medical Subject Headings (MeSH) terms and free-text keywords. Key search terms included “artificial intelligence,” “deep learning,” “machine learning,” and “polycystic ovary syndrome,” among others. These terms, which have been employed in other systematic reviews, were adopted and appropriately adapted for our search strategy. The search for this systematic review was conducted from database inception to January 26, 2026 (**Supplementary Data 1**). The search was performed without any language restrictions or filters on the respective platforms.

It is worth noting that for consistency with the current nomenclature, all included studies originally using the term “PCOS” were mapped to the PMOS construct. Although diagnostic frameworks varied (Rotterdam 2003, NIH criteria, and 2023 International Guidelines), all studies targeted the same clinical entity. Besides, although the initial electronic search was performed without language restrictions to maximize sensitivity, the final inclusion was limited to English-language publications (or those with English full-text translations) because we lacked the resources for reliable translation and data extraction from non-English articles. This approach is consistent with standard practice in diagnostic test accuracy systematic reviews.

### 2.3 PICOTS framework

#### 2.3.1 Population (P)

Reproductive-aged women suspected of having PMOS/PCOS or at risk, based on clinical presentation (menstrual irregularities, hyperandrogenism, or ultrasound features).

#### 2.3.2 Index test (I)

Any ML or DL model developed for binary classification of PMOS/PCOS versus non-PMOS, using any data modality (clinical parameters, biochemical markers, ultrasound imaging, or combinations thereof).

#### 2.3.3 Comparator (C)

Not applicable for this diagnostic accuracy review, as most studies did not compare ML/DL models directly against clinician diagnosis in a head-to-head fashion; instead, we assessed model performance against the reference standard.

#### 2.3.4 Target condition (T)

PMOS, diagnosed according to any recognized diagnostic criteria (Rotterdam 2003, NIH, or 2023 International Guideline, etc.).

#### 2.3.5 Outcome (O)

Outcomes include diagnostic accuracy metrics, including sensitivity, specificity, positive and negative predictive values, area AUC, and diagnostic odds ratio (DOR).

#### 2.3.6 Setting (S)

Settings include any clinical or research setting, including single-center hospitals, multicenter networks, and public database-derived studies.

### 2.4 Inclusion and exclusion criteria

#### 2.4.1 Inclusion criteria

This study included case-control, cohort, and cross-sectional studies that developed DL or ML models for the diagnosis of PMOS and were published in English or had full-text English translations available with extractable data.

#### 2.4.2 Exclusion criteria

Exclusion criteria comprised: duplicate data; studies that did not report statistical metrics and for which data could not be obtained after contacting the authors; AI models other than DL or ML; reviews; conference abstracts; editorials; case reports; study protocols; and not peer-reviewed study.

### 2.5 Data selection and extraction

Duplicate records were manually removed using the duplicate identification strategy in EndNote X7. Titles and abstracts were screened to remove obviously irrelevant studies. The full texts of the remaining articles were then retrieved and thoroughly reviewed to determine eligibility for inclusion in the systematic review. Prior to data extraction, a standardized electronic data extraction form was developed. The extracted information encompassed four main categories to ensure a comprehensive appraisal of each study’s methodology, results, and relevance for clinical interpretation. 1) Study characteristics: first author, year of publication, country of origin, study design, patient source, diagnostic criteria, total sample size, and detailed baseline characteristics of the cohort. 2) Methodological quality and risk of bias: key items influencing the validity of the findings were extracted. These included the method of dataset partitioning (e.g., random split, temporal, or external validation), procedures for feature selection and hyperparameter tuning, and the reporting of model calibration. Adherence to reporting guidelines and availability of code/data for reproducibility were also recorded. 3) Model development and performance: this included model type, specific algorithms used, predictor variables, specificity, sensitivity, accuracy, precision, AUC, F1-score, confusion matrix. Details on the validation set size and generation method were extracted. For studies that provided multiple contingency tables based on different classifier algorithms, datasets, or processing strategies, these were treated as mutually independent. Among studies reporting multiple contingency tables resulting from different preprocessing strategies, only the result with the best performance was selected. If no preprocessing strategy demonstrated clearly superior performance, each strategy was recorded as a separate study, and the corresponding results were collected. 4) Additional contextual information: details on the reference standard application (including blinding), the definition of control groups, funding sources, and declarations of conflicts of interest were collected to inform the interpretation and generalizability of the results.

Two investigators (HYF and SFC) independently performed the literature screening and data extraction. Upon completion, their findings were cross-verified. Any discrepancies were resolved through consultation with a third reviewer (PW). For studies with incomplete published data, we contacted the corresponding authors. Complete confusion matrices were obtained from two studies (Rona et al. and Umaa Maheswari et al.), which were included in the final quantitative synthesis. No other unpublished data were used.

### 2.6 Risk of bias assessment

Two independent reviewers (HYF and FYC) assessed the risk of bias of the included studies using the Quality Assessment of Diagnostic Accuracy Studies-2 (QUADAS-2). QUADAS-2 is a tool designed to evaluate the risk of bias and concerns regarding applicability in diagnostic accuracy studies. It assesses four key domains: patient selection, index test, reference standard, and flow and timing. After the quality assessment, the results were cross-checked. Any disagreements were resolved by consulting a third researcher (PW).

### 2.7 Statistical analysis

This meta-analysis was performed using R version 4.5.2 and STATA 17.0. Pooled estimates of sensitivity, specificity, and diagnostic odds ratio (DOR) with their 95% confidence intervals (CIs) were calculated using a random-effects model, and the Hartung-Knapp-Sidik-Jonkman (HKSJ) method was applied to account for high heterogeneity (27). Hierarchical summary receiver operating characteristic (HSROC) curves were constructed to compare the AUC. The HSROC curve synthesizes diagnostic performance data across studies, taking into account the correlation between sensitivity and specificity within studies and the variation in test accuracy between studies. These curves were estimated from the numbers of true positives, false positives, true negatives, and false negatives from each included study using nonlinear generalized mixed models (28), and the summary AUC was then computed. Heterogeneity was assessed using Cochran’s Q test and quantified by the I^2^ statistic. To quantify the real-world implications of heterogeneity, we calculated 95% prediction intervals (29). Small-study effects were evaluated through Egger’s linear regression test. Additionally, meta-regression was performed to further explore the impact of study-level characteristics (e.g., model type, data source, modeling variables) on diagnostic accuracy, using the log DOR as the outcome variable fitted with a mixed-effects model. To visually demonstrate differences in diagnostic performance across conditions, subgroup analyses were conducted based on model type, data source, and modeling variables. Finally, sensitivity analyses were conducted to assess the robustness of the findings. Outlier studies contributing substantially to overall heterogeneity (>30%) but with low influence on the pooled effect size (<30%) were identified using Baujat plots. After excluding these outliers, the meta-analysis was repeated to evaluate changes in the pooled estimates.

## 3 Results

### 3.1 Study selection

**Figure 1:**
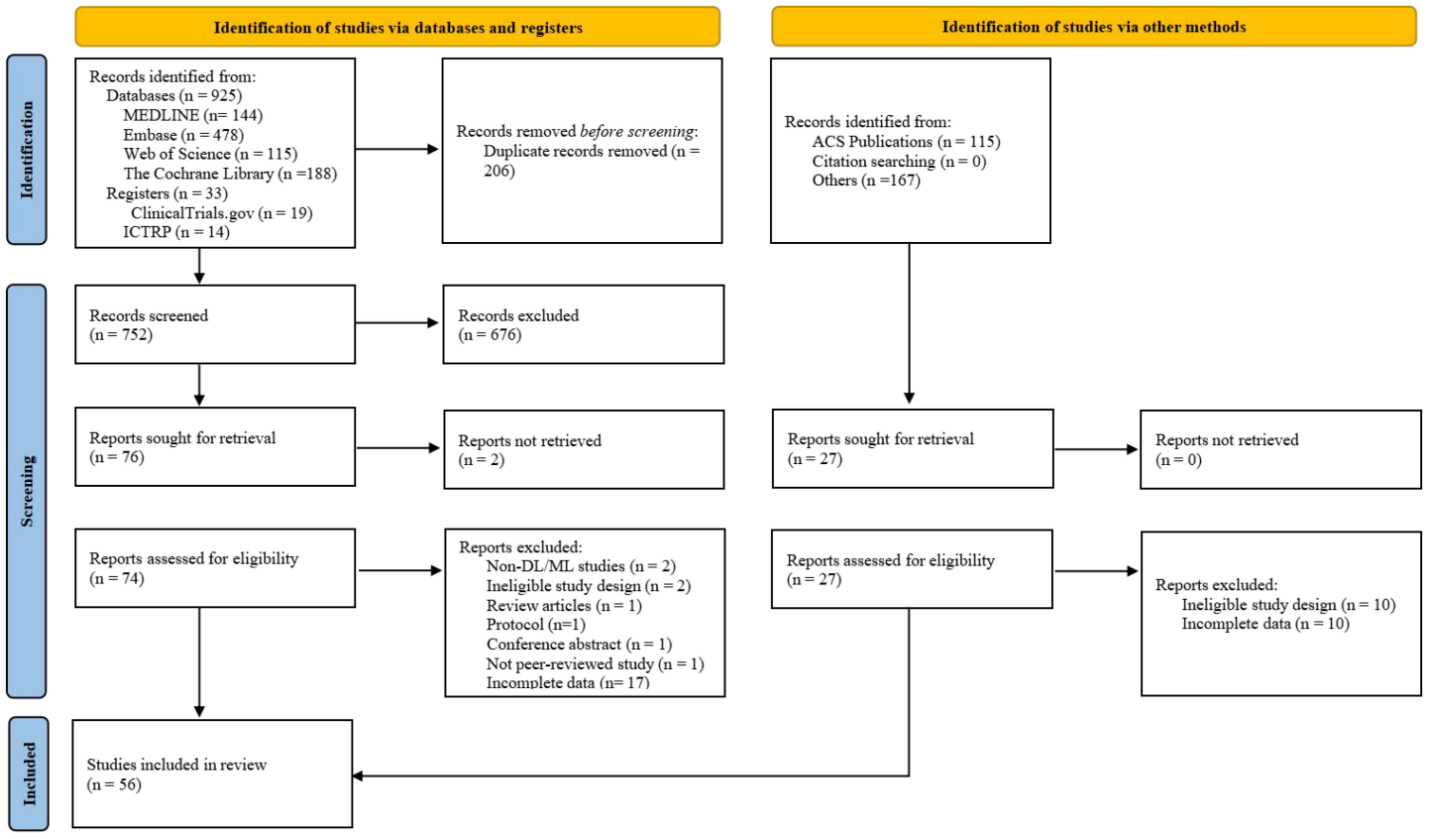
PRISMA 2020 flow diagram of study selection process for the systematic review and meta-analysis of machine learning (ML) and deep learning (DL) models for diagnosing Polyendocrine Metabolic Ovarian Syndrome (PMOS). A total of 1,207 records were identified through database searching (MEDLINE, Web of Science, Embase, Cochrane Library) and other sources (clinical trial registries, gray literature, citation tracking) up to January 26, 2026. After removal of duplicates and screening, 56 studies were included in the final meta-analysis. Studies were excluded due to being non-DL/ML, not meeting study design criteria, being reviews, protocol or conference abstracts, not peer-reviewed study, or having incomplete data. ICTRP, WHO International Clinical Trials Registry Platform; ACS, American Chemical Society; DL, deep learning; ML, machine learning.

A total of 1240 studies were initially included in this study. After duplicate removal, 206 redundant records were excluded, and 676 studies were further excluded by reviewing titles and abstracts. Thus, 103 studies were retained for full-text assessment. After downloading and reviewing the full texts of these 103 studies, 2 studies cannot be retrieved and 45 were excluded for the following reasons: two were not DL/ML related studies, 12 studies that did not meet the research design criteria for inclusion, one review article, one protocol, one conference abstract, one non-peer-reviewed article and 27 had incomplete data with no response from authors upon request. Ultimately, 56 studies (30–84) were included in the final analysis (**Figure 1**).

### 3.2 Study characteristic

All the included studies were conducted between 2019 and 2026. The study designs were predominantly retrospective (n=53) (30–44, 46–64, 66–76, 78–84), with a smaller number of prospective studies (n=3) (45, 65, 77). Geographically, the studies were conducted across 14 countries, with the majority originating from China (n=11) (44–47, 74, 77–81, 83)and India (n=22) (38–40, 42, 43, 48–50, 52–54, 56, 57, 60, 61, 64, 66, 68, 71–73, 76). Other contributing countries included the United States (n=5) (30, 34, 41, 82, 84), Bangladesh (n=4) (55, 69, 70, 75), Saudi Arabia (n=3) (32, 33, 36), Turkey (n=3) (35, 58, 59), Brazil (n=1) (65), England (n=1) (51), and Pakistan (n=1) (62).Some studies were collaborative efforts involving multiple countries, including one from India and Korea (52), two from India and Saudi Arabia (31, 67), one from India, Saudi Arabia with Ethiopia (63), and one from Iraq and Australia (37). Participant data were sourced from various origins. A significant portion of studies utilized public datasets, primarily from Kaggle (n=34) (30, 31, 35–43, 48, 50, 52–57, 60, 62–64, 66, 68, 70–73, 75, 76, 84). Other common sources included hospital-based electronic medical records or clinical data warehouses and outpatient/reproductive medicine clinics (32–34, 44–47, 49, 58, 59, 61, 65, 67, 69, 74, 77–83). For the diagnosis of PMOS, the most frequently applied criterion was the Rotterdam 2003 standard (n=20) (32, 34, 46, 48, 52, 59, 61, 63, 65, 67, 69, 72, 74, 75, 78–81, 83, 84). Several studies did not explicitly state their diagnostic criteria (n=30) (31, 33, 35–41, 43, 44, 47, 49–57, 60, 62, 64, 66, 68, 70, 71, 73, 76), while others employed ultrasonographic findings (49, 67), Chinese guidelines (45), ICD codes combined with Rotterdam 2003 standard (82), the 2023 International Evidence-Based Guideline (30), or labeling by radiology specialists (69). Regarding funding and declarations, a diverse range of sources was reported, including national research grants (e.g., from China, India, USA, Brazil), university funds, and hospital support (31–34, 36–39, 41, 44–48, 51, 55, 56, 61, 63–69, 71, 73, 77–80, 83, 84). 12 studies explicitly declared receiving no external funding (35, 42, 43, 49, 52, 53, 58–60, 70, 74, 76). Notably, one study reported a potential conflict of interest related to industry employment (80). The majority of studies declared no conflicts of interest, while this information was not reported in two studies (39, 64). Prospective registration of the study protocol was rarely mentioned; only one study stated it was “not applicable,” (45) and the remaining studies did not provide registration details. A few studies employed more rigorous designs, such as prospective or multicenter approaches (45, 46, 81), which may enhance generalizability. Furthermore, several studies utilized large-scale or multi-source datasets, contributing to potentially more robust model development and validation (**Supplementary Data 2**).

The methodologies for model development and validation across the included studies demonstrated diverse approaches in dataset partitioning, algorithm selection, feature utilization, and validation strategies. The majority of studies employed an internal validation framework (30–40, 42, 43, 45–47, 49–80, 82–84), with only four studies conducting external validation using independent cohorts (41, 44, 48, 81). The most common method for splitting data into training and testing sets was a random split, typically at ratios of 70:30 or 80:20 (n=14) (30, 40, 41, 50, 52–55, 57, 67, 70, 72, 75, 81). k-fold cross-validation (particularly 5-fold or 10-fold) was also widely used to ensure robustness and reduce overfitting, with some studies applying stratified sampling to address class imbalance (n=28) (35–39, 42–49, 51, 52, 58, 59, 64, 65, 68, 69, 74, 77–80, 82, 84). In several studies, the training and test sets were predefined or generated via hold-out validation (n=3) (32, 61, 73). One study used train-test split (56). 10 studies did not report the validation strategies (31, 33, 34, 60, 62, 63, 66, 71, 76, 83). A wide array of ML and DL algorithms were implemented. Traditional ML models such as Random Forest (RF), Support Vector Machine (SVM), XGBoost, and Logistic Regression (LR) were frequently used, often in comparative or ensemble frameworks (30, 34–38, 40, 42–46, 49, 51, 55–60, 65, 68, 70, 73, 74, 76–84). DL approaches, including Convolutional Neural Networks (CNN) like VGG, ResNet, DenseNet, and custom architectures, were predominantly applied in studies using ultrasound or other image data (31, 32, 39, 41, 47, 50, 66, 67, 72). Ensemble methods and hybrid models were common to enhance predictive performance (33, 48, 52–54, 61–64, 69, 71, 75). Modeling variables spanned multiple data modalities, which contained clinical/demographic, biochemical: hormone levels (LH, FSH, AMH, testosterone, insulin), ultrasound imaging: follicle count, ovarian volume, textural radiomic features, and other including pulse wave analysis, tongue inspection features, and gene expression data. Feature selection was a critical step in many studies to improve model interpretability and performance. Commonly employed techniques included LASSO regression, Recursive Feature Elimination (RFE), Principal Component Analysis (PCA), and various filter methods (e.g., Pearson correlation, Mutual Information). More advanced or hybrid selection methods, such as Boruta, Genetic Algorithms (GA), and Salp Swarm optimization, were also utilized. Notably, a subset of studies, particularly those relying on DL for image analysis, did not employ explicit feature selection, instead leveraging the automated feature extraction capabilities of convolutional networks. And only two studies reported the model calibration (78, 81) (**Supplementary Data 3**).

An assessment of the methodological quality and transparency of the included studies revealed significant concerns across key domains. The application of blinding in the establishment of the gold standard diagnosis was notably absent in the vast majority of studies. Only one study reported partial blinding, where radiologists performing ovary segmentation on MRI were blinded to the patient’s diagnosis (59). Adherence to established ML reporting guidelines was infrequently reported. Only three studies explicitly stated following such guidelines: two studies by Lim et al. followed TRIPOD (45, 46), and Pulluraparbil et al. followed the TRIPOD-AI checklist (54). Regarding transparency and reproducibility, code and data accessibility presented a mixed picture. Data accessibility was relatively high, primarily driven by the extensive use of public datasets like Kaggle and gene expression repositories. For studies using proprietary clinical data, access was typically conditional upon request. In stark contrast, code availability was poor; the overwhelming majority of studies did not provide public access to their analysis scripts or software implementations, which poses a substantial barrier to independent verification and reproducibility (**Supplementary Data 4**).

### 3.3 Methodological quality

**Figure 2:**
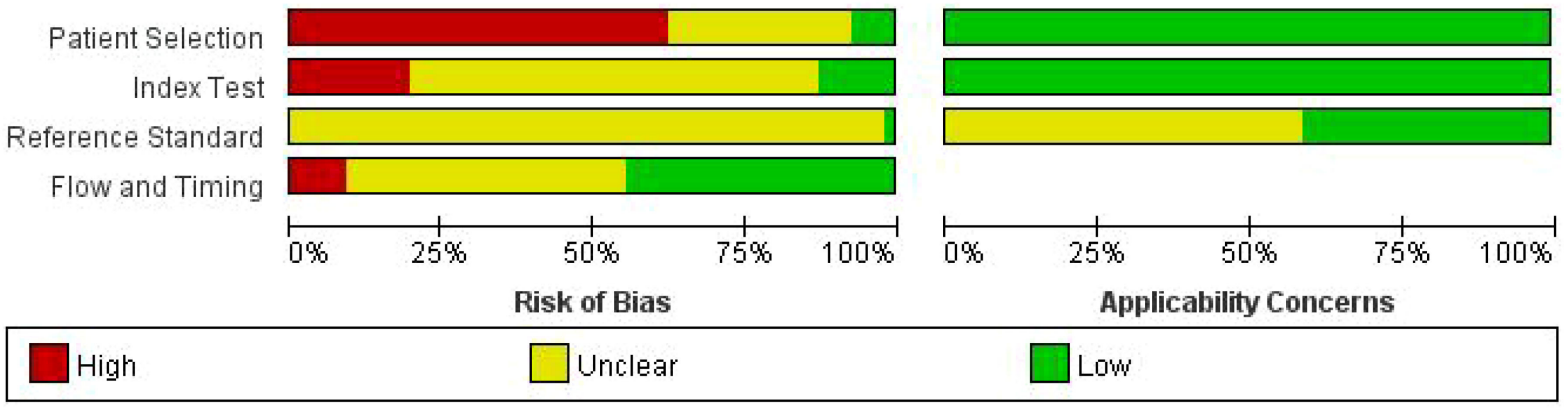
Methodological quality assessment of the 56 included studies using the Quality Assessment of Diagnostic Accuracy Studies-2 (QUADAS-2) tool. The figure shows the proportion of studies with low, high, or unclear risk of bias across the four domains (patient selection, index test, reference standard, flow and timing), and the proportion of studies with low, high, or unclear concerns regarding applicability for the first three domains.

The quality assessment of the included studies was performed using the QUADAS-2 in Review Manager 5.3, with the results presented in **Figure 2** and **Supplementary Figure 1**. In the patient selection domain, 35 studies were assessed as having a high risk of bias because they were case-control studies. All 56 studies were rated as low concern regarding applicability, as there were no significant differences in demographic characteristics or study settings among these studies. In the index test domain, 11 studies were judged as having a high risk of bias due to threshold optimization (45, 46, 53, 60, 65, 66, 69, 73, 74, 80, 84). All 56 studies were rated as low concern regarding applicability. In the reference standard domain, 55 studies were evaluated as having an unclear risk of bias because the blinding in gold standard was unclear (30–58, 60–84). 32 studies they did not specify the diagnostic criteria, making it uncertain whether the method correctly distinguished PMOS (31, 33, 35–44, 47, 50–56, 60, 62–64, 66, 68–73, 76). The applicability concern for these 32 studies was also rated as unclear, as they failed to clearly report the diagnostic criteria used (31, 33, 35–44, 47, 50–56, 60, 62–64, 66, 68–73, 76). In the flow and timing domain, five studies were considered to have a high risk of bias due to missed cases (32, 45, 46, 59, 80). Another 26 studies were rated as having an unclear risk of bias because it was not described whether all patients received the same reference standard (31, 35–39, 41–44, 47, 48, 51, 52, 55, 56, 60, 63, 64, 66, 68, 70, 71, 73, 74, 76).

### 3.4 Performance of ML and DL models for PMOS

**Figure 3:**
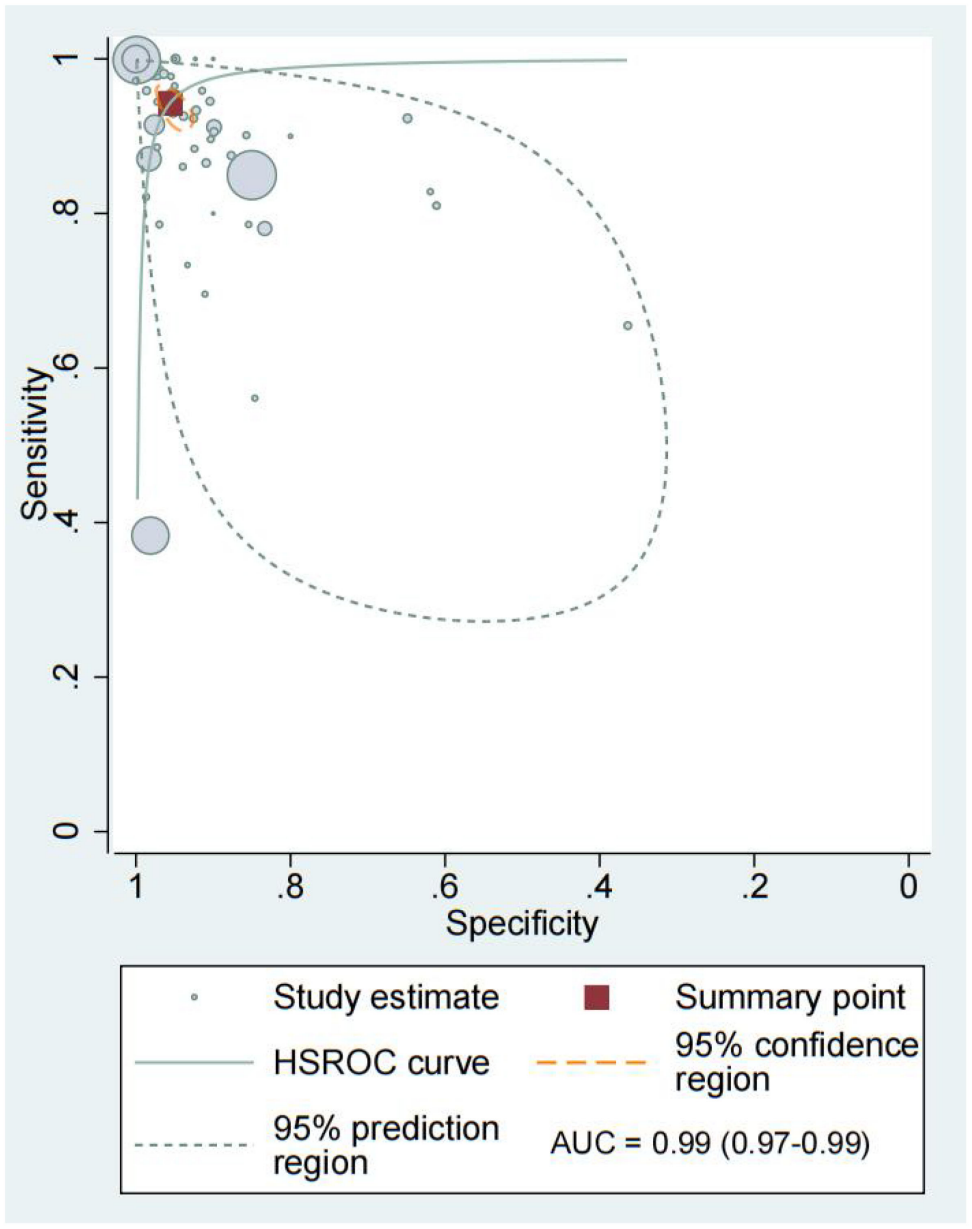
Hierarchical summary receiver operating characteristic (HSROC) curve for machine learning (ML) and deep learning (DL) models in diagnosing Polyendocrine Metabolic Ovarian Syndrome (PMOS). The curve was constructed using data from 60 datasets (56 studies). The area under the HSROC curve was 0.99 (95% CI: 0.97-0.99). The HSROC model accounts for both within-study correlation between sensitivity and specificity and between-study heterogeneity. Each circle represents an individual dataset, with circle size proportional to study sample size.

**Figure 4:**
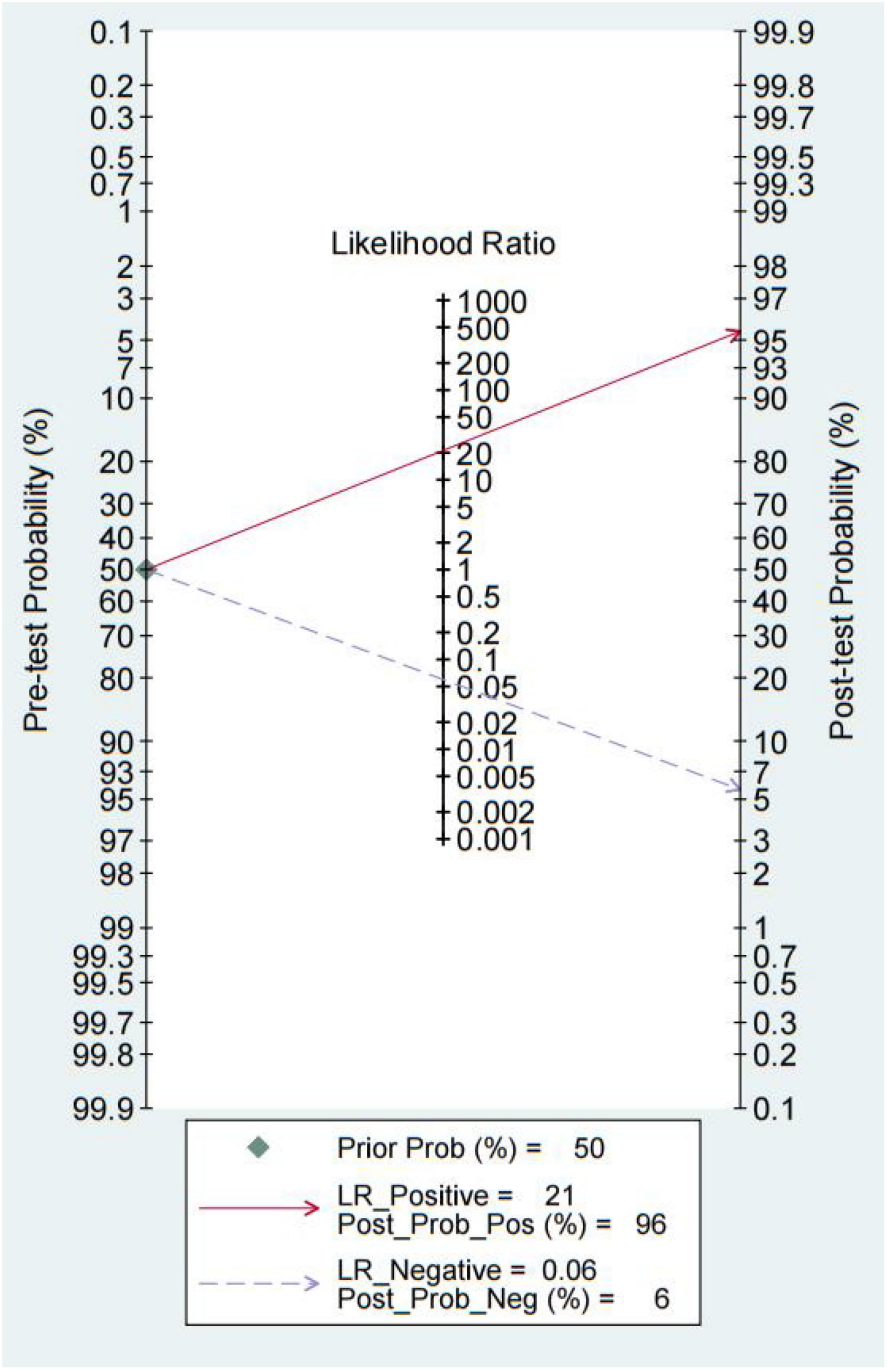
Fagan nomogram for the clinical application of machine learning (ML) and deep learning (DL) models in diagnosing Polyendocrine Metabolic Ovarian Syndrome (PMOS). Based on a pretest probability of 50%, the positive likelihood ratio yields a post-test probability of 96%, meaning that 96% of patients diagnosed with PMOS by the model truly have the disease. The negative likelihood ratio yields a post-test probability of 6%, indicating that 6% of patients classified as non-PMOS by the model actually have the disease.

A total of 60 datasets with the number of cases ranging from 28 to 39,093 (**Supplementary Data 5**) evaluated the diagnostic value of ML or DL models for PMOS. The pooled sensitivity was 0.92 (95% CI: 0.89-0.94, **Supplementary Figure 2**), and specificity was 0.94 (95% CI: 0.91-0.96, **Supplementary Figure 3**). The diagnostic odds ratio was 202.13 (95% CI: 100.97-404.67, **Supplementary Figure 4**). The HSROC curve (**Figure 3**) showed an area under the curve of 0.99 (95% CI: 0.97-0.99) for ML or DL models in diagnosing PMOS. We set the pretest probability to 50% based on the predicted prevalence of the disease. Under this condition, when patients were diagnosed with PMOS by the ML or DL model, the true positive rate accounted for 96%; when the diagnosis was not PMOS, the false negative rate accounted for 6% (**Figure 4**).

### 3.5 Heterogeneity assessment and publication bias

Heterogeneity among the included studies was assessed using Cochran’s Q test and quantified by the *I*^2^ statistic. For sensitivity, substantial heterogeneity was observed (Cochran’s Q = 922.68, df = 59, *P* < 0.001; *I^2^* = 93.6%, 95% CI: 92.4%-94.6%). Similarly, specificity showed high heterogeneity (Cochran’s Q = 890.96, df = 59, *P* < 0.001; *I^2^* = 93.4%, 95% CI: 92.2%-94.4%). To quantify the real-world implications of heterogeneity, we calculated 95% prediction intervals. The prediction interval for sensitivity ranged from 50% to 99% (**Supplementary Figure 2**), and for specificity from 53% to 100% (**Supplementary Figure 3**). This indicates that while the average performance is moderate, the true diagnostic accuracy in a future clinical setting could vary widely.

Small study effect was assessed using Egger’s linear regression test. For sensitivity, the Egger test revealed a statistically significant intercept (intercept = 4.18, 95% CI: 2.71 to 5.65, t = 5.61, *P* < 0.001), indicating the presence of small-study effects or potential publication bias. Similarly, for specificity, the Egger’s linear regression test also showed a significant result (intercept = 1.93, 95% CI: 0.78 to 3.08, t = 3.29, *P* = 0.002), suggesting possible publication bias. These findings suggest that smaller studies with larger effect sizes may have been preferentially published, potentially overestimating the pooled diagnostic accuracy.

### 3.6 Meta-regression

To explore potential sources of heterogeneity, meta-regression was performed using a mixed-effects model with log DOR as the outcome variable. Covariates included model type (ML, DL, ML plus DL), data source (single center, multicenter, database), and modeling variables (clinical parameters, ultrasound image features, clinical plus ultrasound). In univariate analyses, compared to ML models, both DL (coefficient = 2.12, 95% CI: 0.78 - 3.46, *P* = 0.002) and ML+DL models (coefficient = 2.68, 95% CI: 1.44 - 3.92, *P* < 0.001) showed significantly higher diagnostic accuracy. Studies using database data had significantly higher log DOR than those using single-center data (coefficient = 2.13, 95% CI: 0.96 - 3.30, *P* < 0.001), while multicenter studies did not differ significantly (*P* = 0.889). Models based on ultrasound image features alone demonstrated significantly higher accuracy than those based on clinical parameters alone (coefficient = 3.24, 95% CI: 1.93 - 4.56, *P* < 0.001), whereas combined clinical plus ultrasound models showed no significant difference (*P* = 0.779). In multivariate analysis adjusting for all covariates, data source (database vs. single center: coefficient = 1.91, 95% CI: 0.69 - 3.14, *P* = 0.002) and modeling variables (ultrasound image vs. clinical parameters: coefficient = 2.43, 95% CI: 0.66 - 4.20, *P* = 0.007) remained independent predictors of higher diagnostic accuracy. However, the effects of model type were attenuated and no longer statistically significant (DL: *P* = 0.287; ML+DL: *P* = 0.091), suggesting that the apparent advantage of DL and ML+DL models in univariate analyses may be confounded by data source and modeling variables. The full model explained 30.90% of the between-study heterogeneity (R^2^ = 30.90%) (**Table 1**).

**Table 1:** Meta-regression analyses of factors influencing the diagnostic accuracy of machine learning (ML) and deep learning (DL) models for Polyendocrine Metabolic Ovarian Syndrome (PMOS).

Covariate	Level	Univariate analysis		Multivariate analysis	
		coefficient (95% CI)	*P*-value	coefficient (95% CI)	*P*-value
Model type	ML	Reference		Reference	
	DL	2.12 (0.78, 3.46)	0.002	0.99 (-0.84, 2.83)	0.287
	ML+DL	2.68 (1.44, 3.92)	<0.001	1.44 (-0.23, 3.11)	0.091
Data source	Single center	Reference		Reference	
	Multicenter	-0.14 (-2.09, 1.82)	0.889	-0.52 (-2.56, 1.52)	0.618
	Database	2.13 (0.96, 3.30)	<0.001	1.91 (0.69, 3.14)	0.002
Modeling variables	Clinical parameters	Reference		Reference	
	Ultrasound image	3.24 (1.93, 4.56)	<0.001	2.43 (0.66, 4.20)	0.007
	Clinical+Ultrasound	0.23 (-1.40, 1.87)	0.779	-0.82 (-3.00, 1.35)	0.458
Proportion of heterogeneity explained		R^2^		30.90%	

The analysis included 60 datasets from 56 studies. Univariate and multivariate meta-regression were performed using a mixed-effects model with log diagnostic odds ratio (log DOR) as the outcome variable. Covariates included model type (ML, DL, ML+DL), data source (single center, multicenter, database), and modeling variables (clinical parameters, ultrasound image features, clinical plus ultrasound). Coefficients represent the change in log DOR relative to the reference category. The multivariate model explained 30.90% of the between-study heterogeneity (R^2^ = 30.90%). CI, confidence interval; ML, machine learning; DL, deep learning.

### 3.7 Subgroup analysis

Subgroup analyses were performed to visually examine the diagnostic performance of machine learning models for PMOS across different study-level characteristics, focusing on data source and modeling variables, which were identified as independent predictors in meta-regression. For data source, studies using database data demonstrated the highest diagnostic accuracy, with pooled sensitivity of 0.95 (95% CI: 0.92-0.97) and specificity of 0.96 (95% CI: 0.93-0.98), followed by multicenter studies (sensitivity: 0.88, 95% CI: 0.59-0.98; specificity: 0.85, 95% CI: 0.41-0.98) and single-center studies (sensitivity: 0.84, 95% CI: 0.74-0.90; specificity: 0.90, 95% CI: 0.85-0.94). Regarding modeling variables, models based solely on ultrasound image features achieved the highest accuracy (sensitivity: 0.99, 95% CI: 0.97-1.00; specificity: 0.98, 95% CI: 0.93-1.00), outperforming those based on clinical parameters alone (sensitivity: 0.91, 95% CI: 0.87-0.93; specificity: 0.93, 95% CI: 0.90-0.95). Models combining clinical and ultrasound features showed intermediate performance (sensitivity: 0.87, 95% CI: 0.55-0.93; specificity: 0.95, 95% CI: 0.86-0.98). Subgroup analysis by model type revealed that ML+DL and DL models had slightly higher point estimates than ML models, but the differences were not statistically significant, consistent with the meta-regression findings (all *P* > 0.05, **Table 1**). Detailed results are summarized in **Table 2**.

**Table 2:** Subgroup analysis of diagnostic accuracy for machine learning (ML) and deep learning (DL) models in Polyendocrine Metabolic Ovarian Syndrome (PMOS) diagnosis.

	Dataset, n	Pooled sensitivity (95% CI)	I^2^ (%) for sensitivity	Pooled specificity (95% CI)	I^2^ (%) for specificity
Model type					
ML	37	0.88 (0.84-0.92)	92.3	0.92 (0.88-0.94)	92.8
DL	10	0.97 (0.88-0.99)	93.1	0.97 (0.91-0.99)	88.9
DL+ML	13	0.97 (0.93-0.98)	86.5	0.98 (0.94-0.99)	89.8
Source of data					
single center	16	0.84 (0.74-0.90)	94.4	0.90 (0.85-0.94)	95.6
multicenter	5	0.88 (0.59-0.98)	72.2	0.85 (0.41-0.99)	86.4
Database	35	0.95 (0.92-0.97)	88.1	0.96 (0.93-0.98)	87.1
Modeling variables					
clinical parameters	37	0.91 (0.87-0.93)	93.0	0.93 (0.90-0.95)	93.3
ultrasound image	12	0.99 (0.97-1.00)	90.5	0.98 (0.93-1.00)	91.1
clinical + ultrasound	6	0.87 (0.55-0.93)	73.8	0.95 (0.86-0.98)	84.2

The analysis includes 60 datasets from 56 studies, stratified by model type, data source, and modeling variables. Pooled sensitivity and specificity with 95% confidence intervals (CIs) were estimated using random-effects models with the Hartung-Knapp-Sidik-Jonkman (HKSJ) method. Heterogeneity within each subgroup is quantified using the I^2^ statistic. The number of datasets (n) in each subgroup is indicated. Subgroup analyses were conducted to visually explore sources of heterogeneity identified in meta-regression. CI, confidence interval; ML, machine learning; DL, deep learning.

### 3.8 Sensitivity analysis

We performed outlier and influence analysis to detect studies contributing to heterogeneity and affecting the pooled effect size, as measured by the Baujat plot. Based on the results shown in the Baujat plot (**Supplementary Figure 5**), we identified the study Lim 2023 as an influential outlier and performed meta-analysis of sensitivity, specificity, and DOR on the remaining 59 datasets. The results are presented in **Supplementary Figure 6**.

## 4 Discussion

This meta-analysis comprehensively evaluated the diagnostic performance of ML or DL models in identifying PMOS. Based on 56 studies comprising 60 datasets, the pooled sensitivity and specificity were 0.92 (95% CI: 0.89-0.94) and 0.94 (95% CI: 0.91-0.96), respectively, with a HSROC area under the curve of 0.99 (95% CI: 0.97-0.99). At a pretest probability of 50%, the positive post-test probability was 96%, while the negative post-test probability was 6%. However, these seemingly impressive summary estimates must be interpreted against a backdrop of a remarkably limited evidence base: only 6 of the 60 included dataset had sample sizes exceeding 1,000 cases. Small-sample studies are inherently prone to overfitting and have limited statistical power, raising concerns about whether the reported high accuracies can be replicated in larger, more diverse populations. AI diagnostic algorithms, particularly DL models, fundamentally require large-scale, diverse datasets for robust training and validation (85–87). More critically, substantial heterogeneity was observed across studies (sensitivity *I*^2^=93.6%, specificity *I*^2^=93.4%), and the 95% prediction intervals were extremely wide (sensitivity: 50%-99%; specificity: 53%-100%). This indicates that while the average performance is high, the true diagnostic accuracy in any future clinical setting could vary dramatically. These summary estimates should therefore be viewed not as precise predictions for any individual model, but rather as a central tendency across a highly variable body of literature. The 95% prediction intervals-ranging from 50% to 99% for sensitivity and 53% to 100% for specificity-serve as a stark reminder that the true performance of any single model deployed in a future clinical setting could be substantially lower than the pooled average suggests. This wide variability underscores the urgent need for rigorous external validation and should temper overoptimistic interpretations of the summary estimates.

The vast majority of included studies employed single-center designs, relying on data from a single institution or specific population. Subgroup analysis revealed that single-center studies had significantly lower diagnostic performance (pooled sensitivity 0.84, specificity 0.90) compared to database-derived studies (sensitivity 0.95, specificity 0.96). This performance gap underscores the inherent limitations of single-center designs: models may excel in the specific population on which they were trained, but their performance can deteriorate sharply when applied to different geographic regions, ethnicities, medical devices, or clinical practices. Such selection bias and population specificity severely constrain model generalizability. Achieving true clinical translation will require breaking down “data silos” and establishing multi-center, cross-regional data sharing and collaboration frameworks. Specific priorities include: 1) developing national or international PMOS imaging and clinical databases with standardized data acquisition and annotation protocols; 2) adopting privacy-preserving technologies such as federated learning to enable collaborative modeling without sharing raw data; and 3) requiring data sharing policies from funding agencies and journals to facilitate independent validation and iterative model refinement. It is noteworthy that only 5 datasets in this review were multi-center, and their pooled performance did not significantly differ from single-center studies (P = 0.889), possibly reflecting the still-limited sample sizes and uncontrolled inter-center heterogeneity in these studies.

The most critical barrier to clinical translation identified in this review is the near-complete absence of external validation: only 4 studies (7.1%) evaluated their models on truly independent, external cohorts. This means that the high diagnostic accuracies reported in the literature more likely reflect model performance under “optimal conditions” rather than their predictive ability in real-world clinical settings. Excellent performance on development data may arise from overfitting to noise, the “idealized” samples introduced by case-control designs, or inadvertent data leakage during feature selection or threshold optimization. Consequently, the summary accuracy estimates from this meta-analysis should be cautiously interpreted as estimates of “upper-bound potential” rather than guarantees of real-world clinical effectiveness. Future research must prioritize external validation as an essential component of model development from the outset. Models should be evaluated for stability across multiple dimensions: 1) different geographic regions and ethnic populations; 2) different ultrasound device manufacturers and models; 3) operators with varying levels of experience; and 4) different clinical settings (e.g., tertiary hospitals vs. primary care clinics). Only through multi-layered, multi-dimensional external validation can we determine whether a model truly possesses the robustness required for clinical deployment.

Subgroup analysis revealed that models based solely on ultrasound image features achieved the highest diagnostic accuracy (pooled sensitivity 0.99, pooled specificity 0.98), substantially outperforming models based on clinical parameters alone (sensitivity 0.91, specificity 0.93). This finding has important clinical implications: ultrasound images directly capture one of the core diagnostic criteria for PMOS-polycystic ovarian morphology (PCOM)-and deep learning models excel at automatically extracting complex textural and structural features from high-dimensional image data, potentially detecting subtle patterns imperceptible to the human eye. This offers a technological pathway toward standardizing ovarian assessment, reducing inter-observer variability, and enhancing diagnostic capacity in resource-limited settings. However, the reproducibility of this exceptional performance in real-world settings faces multiple challenges. First, domain shift: variations in ultrasound device manufacturers, probe frequencies, image acquisition parameters, operator techniques, and patient positioning can all alter image distributions, leading to performance degradation. Second, variability in annotation: the definition of PCOM itself involves subjectivity; inter-radiologist variability in follicle counting and ovarian volume measurement introduces label noise that directly impacts model learning. Third, population heterogeneity: ovarian ultrasound characteristics may vary across ethnicities, ages, and BMI categories, limiting the generalizability of models trained on homogeneous populations. Future research must therefore incorporate multi-center, multi-device, multi-operator imaging data into training sets and employ domain generalization or domain adaptation techniques to enhance model robustness to variations in image acquisition conditions. Standardized image acquisition and annotation protocols should be established to minimize operator-induced variability. From a biological perspective, the superior performance of ultrasound-based models likely reflects their direct capture of follicular architecture and ovarian stromal characteristics-key determinants of ovulatory function and reproductive potential that are not fully captured by biochemical markers alone.

While subgroup analyses suggested superior performance for DL models, database-derived datasets, and ultrasound image-based models, these findings must be interpreted cautiously within the limitations of observational comparisons. These comparisons are not based on randomization and are therefore susceptible to multiple confounding factors. First, confounding between model type and data quality: The apparent superiority of DL models in univariate analysis (coefficient 2.12) may not solely reflect algorithmic advantages, but rather their application to larger, better-annotated public datasets (e.g., Kaggle), while traditional ML models were more often applied to smaller, single-center clinical datasets. Multivariate meta-regression supports this interpretation: after adjusting for data source and modeling variables, the effect of model type was attenuated and no longer statistically significant (DL: P = 0.287; ML+DL: P = 0.091), suggesting that the apparent advantage of DL may be confounded by data scale and modality. Second, higher “signal strength” of imaging data: ultrasound images directly contain morphological information about PCOM, whereas clinical parameters (e.g., hormone levels, menstrual history) are only indirect indicators. Image-based models therefore have an inherent advantage in discriminative capacity. Furthermore, researchers may preferentially apply imaging-based modeling to higher-quality datasets, further amplifying this apparent difference. Third, publication bias may exaggerate subgroup differences: Egger’s test revealed significant small-study effects (sensitivity *P* < 0.001, specificity *P* = 0.002), indicating that studies reporting high diagnostic accuracy are more likely to be published, while negative or low-performing studies may be missing. This selective reporting could systematically bias subgroup comparisons. Therefore, these subgroup differences should not be interpreted as causal claims, but rather as trends observed under specific conditions that can inform future research design: direct comparisons of different model types on the same dataset, comparisons of different data modalities within the same population, and prospective designs that control for confounding.

Previous systematic reviews on AI in PMOS, such as those by Barrera et al. (2023) (88), Verma et al. (2024) (17), and Wang et al. (2025) (23), have provided valuable narrative summaries, consistently noting high diagnostic accuracy but also highlighting methodological heterogeneity and inconsistent use of diagnostic criteria. The present study advances this literature by providing the first quantitative synthesis of diagnostic accuracy through meta-analysis, and by systematically exploring sources of heterogeneity through subgroup analysis and meta-regression. Our findings are consistent with prior narrative reviews but further reveal: 1) that while summary diagnostic accuracy is high, the underlying evidence base is remarkably limited; 2) that ultrasound image features and database-derived data are independent predictors of higher accuracy; and 3) that unreported diagnostic criteria and lack of external validation represent the most critical evidence gaps.

Beyond the limitations discussed above, this study has several methodological constraints. First, the QUADAS-2 tool was designed for traditional diagnostic accuracy studies and may not fully capture biases specific to AI research, such as data leakage during preprocessing, the impact of multiple comparisons during hyperparameter optimization, or the lack of model calibration reporting. We acknowledge that these AI-specific biases were not systematically assessed in our review, as a validated AI-tailored quality assessment tool (e.g., QUADAS-AI) was not available at the time of our analysis. Future reviews should employ such dedicated tools to provide a more comprehensive risk-of-bias evaluation (89). Second, some subgroup analyses were based on small numbers of studies (e.g., only 5 multi-center studies, only 6 clinical plus ultrasound modeling studies), warranting cautious interpretation. Third, because calibration was rarely reported in primary studies, we could not assess the accuracy of predicted probabilities, which is essential for clinical decision-making. Last but not least, the failure of over half of the included studies to report their diagnostic criteria introduced a significant and unquantifiable source of bias, limiting our ability to verify that all studies were targeting the same clinical condition. Based on the evidence gaps identified, future research should prioritize: 1) development of large-scale, multi-center, multi-ethnic datasets with standardized data sharing frameworks; 2) mandatory external validation as a prerequisite for model development and publication; 3) adherence to AI-specific reporting guidelines (e.g., TRIPOD-AI) to ensure transparency and reproducibility; 4) integration of explainable AI techniques to illuminate model decision-making and build clinician trust; 5) development of advanced multi-modal fusion models that integrate clinical, biochemical, imaging, and genomic data-an approach that has shown promise in other complex disease areas such as cardiovascular risk prediction and 6) prospective interventional studies-ultimately, randomized controlled trials-to demonstrate whether AI-assisted diagnosis improves patient outcomes.

The diagnosis of PMOS is fundamentally linked to reproductive health outcomes, including anovulatory infertility, menstrual cycle dysfunction, and pregnancy complications. In current practice, ovarian morphology assessment-a cornerstone of PMOS diagnosis under the Rotterdam criteria-relies heavily on subjective ultrasound evaluation, leading to substantial inter-observer variability and diagnostic delays that may adversely affect fertility treatment timelines. AI-assisted analysis of ovarian ultrasound images, as demonstrated in our subgroup analysis (pooled sensitivity 0.99, specificity 0.98), offers a potential solution to standardize the identification of PCOM. This could have several important clinical implications: 1) for infertility evaluation, automated and reproducible PCOM detection could expedite the diagnostic workup for women presenting with anovulatory infertility, reducing time-to-diagnosis and enabling earlier initiation of ovulation induction therapies; 2) for reproductive counseling, more accurate and consistent PMOS diagnosis could improve the precision of prognostic discussions regarding natural conception, response to assisted reproductive technologies, and risks of ovarian hyperstimulation syndrome; 3) for clinical decision-making, AI tools could serve as a triage mechanism, flagging high-risk cases for specialist review and potentially reducing unnecessary referrals, thereby improving healthcare resource allocation in busy reproductive medicine clinics. However, we must emphasize that these potential benefits remain theoretical until prospective studies demonstrate that AI-assisted diagnosis translates into improved patient-relevant outcomes, such as shorter time to pregnancy, higher live birth rates, or reduced treatment-related morbidity.

## 5 Conclusion

This meta-analysis demonstrates that ML and DL models have the potential to achieve promising diagnostic accuracy for PMOS, particularly when leveraging ultrasound imaging and large-scale, diverse datasets. The performance of ultrasound image-based models suggests that AI-assisted analysis of ovarian morphology could eventually standardize and enhance a traditionally subjective component of PMOS diagnosis, potentially reducing inter-observer variability and improving diagnostic consistency across different healthcare settings. However, these promising findings must be balanced against the sobering limitations of the current evidence base. The field is characterized by small samples, single-center designs, inadequate external validation, and most fundamentally widespread failure to report diagnostic criteria. These limitations mean that current AI models are not yet ready for unsupervised clinical use. Rather, they should be viewed as assistive tools that can provide second opinions or prioritize high-risk cases for further evaluation, with their outputs always interpreted within the full clinical context.

The broader implications of this review extend beyond PMOS to the entire field of AI in medicine. Our findings underscore that high reported accuracy is not synonymous with clinical readiness. Without external validation, transparent reporting of reference standards and demonstration of generalizability across diverse populations, even the most impressive summary estimates remain research benchmarks rather than clinically deployable tools. The 95% prediction intervals reported here-ranging from 50% to 99% for sensitivity-serve as a stark reminder that the true performance of any individual model in a future clinical setting could be far lower than current literature suggests.

Achieving clinical translation will require a paradigm shift in how AI diagnostic research is conducted and reported. Future research must prioritize: 1) development of large-scale, multi-center, multi-ethnic datasets with standardized diagnostic criteria and mandatory data sharing; 2) rigorous external validation as a prerequisite for publication; 3) adherence to AI-specific reporting guidelines, such as TRIPOD-AI; 4) integration of explainable AI techniques to build clinician trust; and 5) ultimately, prospective studies and randomized controlled trials demonstrating that AI-assisted diagnosis improves patient outcomes. Only through such methodological rigor can AI-assisted diagnostic tools move from “high accuracy in papers” to “high credibility in practice,” delivering on their promise to enhance the accuracy, efficiency, and accessibility of PMOS diagnosis worldwide.

## Data Availability

The original contributions presented in the study are included in the article/**Supplementary Material**. Further inquiries can be directed to the corresponding authors.
