# Supplementary material for: Machine learning and deep learning for diagnosis of Polyendocrine Metabolic Ovarian Syndrome: systematic review and meta-analysis
Source: Front Endocrinol (Lausanne). 2026 Jul 30;17:1889375. doi: 10.3389/fendo.2026.1889375 (PMC13468022; doi:10.3389/fendo.2026.1889375)
Supplement: Supplementary file 1 [file SupplementaryFile1.docx]

**Supplementary Data 1. Search strategies**

**MEDLINE via PubMed (NCBI)：144**

#1 ("Polycystic Ovary Syndrome"[Mesh]) OR (PCOS[tiab] OR "polycystic ovarian syndrome"[tiab] OR "polycystic ovary syndrome"[tiab] OR "Stein-Leventhal syndrome"[tiab])

#2 ("Machine Learning"[Mesh] OR "Artificial Intelligence"[Mesh] OR "Deep Learning"[Mesh] OR "Neural Networks, Computer"[Mesh] OR "Algorithms"[Mesh]) OR (machine learning[tiab] OR "deep learning"[tiab] OR "artificial intelligence"[tiab] OR "neural network*"[tiab] OR "random forest"[tiab] OR "support vector machine*"[tiab] OR "SVM"[tiab] OR "classification model"[tiab] OR "predictive model"[tiab] OR "diagnostic model"[tiab] OR "computer-assisted diagnosis"[tiab] OR "CAD"[tiab])

#3 ("Diagnosis, Computer-Assisted"[Mesh] OR "Sensitivity and Specificity"[Mesh] OR "ROC Curve"[Mesh] OR "Area Under Curve"[Mesh]) OR (diagnos*[tiab] AND (accuracy[tiab] OR accurate[tiab] OR "ROC"[tiab] OR "AUC"[tiab] OR "area under the curve"[tiab] OR "sensitivity"[tiab] OR "specificity"[tiab] OR "validation"[tiab] OR "validity"[tiab] OR "discrimination"[tiab] OR "calibration"[tiab]))

#4  ((("Polycystic Ovary Syndrome"[Mesh]) OR (PCOS[tiab] OR "polycystic ovarian syndrome"[tiab] OR "polycystic ovary syndrome"[tiab] OR "Stein-Leventhal syndrome"[tiab])) AND (("Machine Learning"[Mesh] OR "Artificial Intelligence"[Mesh] OR "Deep Learning"[Mesh] OR "Neural Networks, Computer"[Mesh] OR "Algorithms"[Mesh]) OR (machine learning[tiab] OR "deep learning"[tiab] OR "artificial intelligence"[tiab] OR "neural network*"[tiab] OR "random forest"[tiab] OR "support vector machine*"[tiab] OR "SVM"[tiab] OR "classification model"[tiab] OR "predictive model"[tiab] OR "diagnostic model"[tiab] OR "computer-assisted diagnosis"[tiab] OR "CAD"[tiab]))) AND (("Diagnosis, Computer-Assisted"[Mesh] OR "Sensitivity and Specificity"[Mesh] OR "ROC Curve"[Mesh] OR "Area Under Curve"[Mesh]) OR (diagnos*[tiab] AND (accuracy[tiab] OR accurate[tiab] OR "ROC"[tiab] OR "AUC"[tiab] OR "area under the curve"[tiab] OR "sensitivity"[tiab] OR "specificity"[tiab] OR "validation"[tiab] OR "validity"[tiab] OR "discrimination"[tiab] OR "calibration"[tiab])))

**Embase via [Embase.com](https://embase.com/) (Elsevier) : 478**

#1 'polycystic ovary syndrome'/exp OR pcos:ti,ab OR 'polycystic ovarian syndrome':ti,ab OR 'polycystic ovary syndrome':ti,ab OR 'stein leventhal syndrome':ti,ab

#2 'machine learning'/exp OR 'artificial intelligence'/exp OR 'deep learning'/exp OR 'neural network'/exp OR 'supervised machine learning'/exp OR 'unsupervised machine learning'/exp OR 'computer assisted diagnosis'/exp OR 'machine learning':ti,ab OR 'deep learning':ti,ab OR 'artificial intelligence':ti,ab OR 'neural network*':ti,ab OR 'random forest':ti,ab OR 'support vector machine*':ti,ab OR svm:ti,ab OR 'classification model':ti,ab OR 'predictive model':ti,ab OR 'diagnostic model':ti,ab OR 'computer assisted diagnosis':ti,ab OR cad:ti,ab

#3 'diagnostic accuracy'/exp OR 'sensitivity and specificity'/exp OR 'receiver operating characteristic'/exp OR 'area under the curve'/exp OR 'validation study'/exp OR 'algorithm'/exp OR ((diagnos* NEAR/3 (accuracy OR accurate OR validation OR validity OR performance OR evaluate*)):ti,ab) OR roc:ti,ab OR auc:ti,ab OR 'area under the curve':ti,ab OR 'c statistic':ti,ab OR 'c-statistic':ti,ab OR sensitivit*:ti,ab OR specificit*:ti,ab OR 'positive predictive value':ti,ab OR 'negative predictive value':ti,ab OR (((discrimination OR calibration) NEAR/3 (model OR analysis)):ti,ab)

#1 AND #2 AND #3

**Web of Science core Collection via Clarivate Analytics (Web of Science) :115**

#1 TS=(PCOS OR "polycystic ovarian syndrome" OR "polycystic ovary syndrome" OR "Stein-Leventhal syndrome")

#2 TS=("machine learning" OR "deep learning" OR "artificial intelligence" OR "neural network*" OR "random forest" OR "support vector machine*" OR SVM OR "classification model" OR "predictive model" OR "diagnostic model" OR "computer assisted diagnosis" OR CAD)

#3 TS=(diagnos* NEAR/3 (accuracy OR accurate OR validation OR validit* OR performance OR evaluat* OR model)) OR (("receiver operating characteristic" OR ROC OR AUC OR "area under the curve" OR "c statistic") NEAR/3 (diagnos* OR model)) OR(sensitivity OR specificity OR "positive predictive value" OR "negative predictive value")

#4 #1AND #2 AND #3

****The Cochrane Library (via Wiley Online Library) :188****

**#1	MeSH descriptor: [Polycystic Ovary Syndrome] explode all trees**

**#2	polycystic ovary syndrome**

**#3	polycystic ovarian syndrome**

**#4	Stein-Leventhal syndrome**

**#5	PCOS**

**#6	#1 OR #2 OR #3 OR #4 OR #5**

**#7	MeSH descriptor: [Machine Learning] explode all trees**

**#8	MeSH descriptor: [Artificial Intelligence] explode all trees**

**#9	MeSH descriptor: [Deep Learning] explode all trees**

**#10	MeSH descriptor: [Neural Networks, Computer] explode all trees**

**#11	MeSH descriptor: [Algorithms] explode all trees**

**#12	machine learning**

**#13	deep learning**

**#14	artificial intelligence**

**#15	neural network***

**#16	random forest**

**#17	support vector machine***

**#18	SVM**

**#19	classification model**

**#20	predictive model**

**#21	diagnostic model**

**#22	computer-assisted diagnosis**

**#23	CAD**

**#24	#7 OR #8 OR #9 OR #10 OR #11 OR #12 OR #13 OR #14 OR #15 OR #16 OR #17 OR #18 OR #19 OR #20 OR #21 OR #22**

**#25	MeSH descriptor: [Diagnosis, Computer-Assisted] explode all trees**

**#26	MeSH descriptor: [Sensitivity and Specificity] explode all trees**

**#27	MeSH descriptor: [ROC Curve] explode all trees**

**#28	MeSH descriptor: [Area Under Curve] explode all trees**

**#29	diagnos***

**#30	accuracy**

**#31	accurate**

**#32	ROC**

**#33	AUC**

**#34	area under the curve**

**#35	sensitivity**

**#36	specificity**

**#37	validation**

**#38	validity**

**#39	discrimination**

**#40	calibration**

**#41	#25 OR #26 OR #27 OR #28 OR #29 OR #30 OR #31 OR #32 OR #33 OR #34 OR #35 OR #36 OR #37 OR #38 OR #39 OR #40**

**#42	#6 AND #24 AND #41**

****[ClinicalTrials.gov](https://clinicaltrials.gov/) (U.S. National Library of Medicine): 19****

**(polycystic ovary OR PCOS) AND (machine learning OR artificial intelligence OR AI OR algorithm)**

****International Clinical Trials Registry Platform: 14****

**(polycystic ovary OR PCOS) AND (machine learning OR artificial intelligence OR AI OR algorithm)**

****ACS Publications: 115****

**(Anywhere): (polycystic ovary syndrome OR PCOS OR “Stein-Leventhal”)**

**AND (Anywhere): (“machine learning” OR “deep learning” OR “artificial intelligence” OR “neural network” OR algorithm* OR “predictive model” OR chemometrics)**
