# Supplementary material for: Machine learning and deep learning for diagnosis of Polyendocrine Metabolic Ovarian Syndrome: systematic review and meta-analysis
Source: Front Endocrinol (Lausanne). 2026 Jul 30;17:1889375. doi: 10.3389/fendo.2026.1889375 (PMC13468022; doi:10.3389/fendo.2026.1889375)
Supplement: Supplementary file 3 [file SupplementaryFile11.docx]

**Supplementary Figure 3.** Forest plot of sensitivity, specificity, and diagnostic odds ratio for the remaining 59 studies after excluding Lim 2023, which was identified as an influential outlier by Baujat plot. The pooled sensitivity was 0.92 (95% CI: 0.89-0.95) with an *I*² of 93.5%; the pooled specificity was 0.94 (95% CI: 0.92-0.96) with an *I*² of 93.2%; the diagnostic odds ratio was 220.49 (95% CI: 111.73-435.13; *I*² = 93.2%).
