# Supplementary material for: Machine learning and deep learning for diagnosis of Polyendocrine Metabolic Ovarian Syndrome: systematic review and meta-analysis
Source: Front Endocrinol (Lausanne). 2026 Jul 30;17:1889375. doi: 10.3389/fendo.2026.1889375 (PMC13468022; doi:10.3389/fendo.2026.1889375)
Supplement: Supplementary file 4 [file SupplementaryFile2.docx]

**Supplementary Data 2. Information of the included studies**

| **Study** | **First author** | **Publication year** | **Country** | **Study type** | **Source of the patient** | **Standard for diagnosis** | **Funding** | **Conflict of interest** | **Registration** |
| --- | --- | --- | --- | --- | --- | --- | --- | --- | --- |
| Cheng 2019 | Jay Jojo Cheng | 2019 | America | Retrospective, single-center | Electronic Medical Records (Clinical Data Warehouse) from Boston Medical Center (inpatients/outpatients) | Rotterdam criteria (2003) | Eunice Kennedy Shriver National Institute of Child Health and Human Development (NICHD), NIH (K12 HD000849) | No conflicts declared | Not mentioned |
| Lv 2022 | Wenqi Lv | 2022 | China | Retrospective, single-center | Outpatient clinic (hospital) and community checkup, Peking University Third Hospital | Not explicitly stated | National Key R&D Program of China, National Natural Science Foundation of China, Tsinghua University Spring Breeze Fund, Beijing Lab Foundation, Tsinghua Autonomous Research Foundation. | No conflicts declared | Not mentioned |
| Zhang 2021 | Xinyi Zhang | 2021 | China | Retrospective, single-center | Outpatient clinic (Center of Reproductive Medicine, Shengjing Hospital of China Medical University) | Rotterdam criteria (2003) | National Key Research and Development Program (2018YFC1002105); CIHR Foundation Scheme Grant. | No conflicts declared | Not mentioned |
| Xie 2022 | Linling Xie | 2022 | China | Retrospective | Public database (GEO) | Rotterdam criteria (2003) | National Natural Science Foundation of China (No. 81804135). | No conflicts declared | Not mentioned. |
| Sreejith 2022 | S. Sreejith | 2022 | India | Retrospective | Public database (Kaggle) | Not explicitly stated | Not mentioned | No conflicts declared | Not mentioned |
| Xu 2022 | Huiyu Xu | 2022 | China | Retrospective, single-center | Reproductive medicine clinic of Peking University Third Hospital (infertility/ART clinic) | Rotterdam criteria (2003) | National Natural Science Foundation of China; National Key R&D Program; Peking University Third Hospital Innovation & Transfer Fund; Major National R&D Projects; Capital Health Research and Development of Special Projec | Yes (KA employed by The Predict Health; YH employed by Hangzhou Qingguo Medical Technology Co. Ltd.) | Not mentioned |
| Suha 2022 | Sayma Alam Suha | 2022 | Bangladesh | Retrospective, multi-center | Ultrasound images from open internet sources and two diagnostic centers and three hospitals in Bangladesh (Combined Military Hospital included) | Labeling by radiology specialists | Information and Communication Technology (ICT) Division, Ministry of Posts, Telecommunications and Information Technology, Bangladesh | No conflicts declared | Not mentioned |
| Mehr 2022 | Homay Danaei Mehr | 2022 | Turkey | Retrospective | Public database (Kaggle) | Not explicitly stated | None declared | No conflicts declared | Not mentioned |
| Zigarelli 2022 | Angela Zigarelli | 2022 | America | Retrospective | Public database (Kaggle) | Not explicitly stated | Not mentioned | No conflicts declared | Not mentioned |
| Silva 2021 | S. Silva | 2021 | Brazil | Prospective, single-center | PCOS patients from the Division of Endocrinology of an academic hospital; healthy controls recruited from university employees and students. | Rotterdam criteria (2003) | Conselho Nacional de Desenvolvimento Científico e Tecnológico (CNPq) | No conflicts declared | Not mentioned |
| Tiwari 2022 | Shamik Tiwari | 2022 | India | Retrospective, multi-center | Public database (Kaggle) | Not explicitly stated | Taif University Researchers Supporting Project (TURSP-2020/114), Taif University, Saudi Arabia | No conflicts declared | Not mentioned |
| Alamoudi 2023 | Abrar Alamoudi | 2023 | Saudi Arabia | Retrospective, single-center | King Fahad Hospital of the University (Khobar, Saudi Arabia), outpatient and inpatient | Rotterdam criteria (2003) | Not mentioned | No conflicts declared | Not mentioned |
| Wang 2023 | Weiying Wang | 2023 | China | Retrospective, single-center | Shuguang Hospital Affiliated to Shanghai University of TCM (PCOS patients from outpatient clinic; healthy controls from Physical Examination Center of the same hospital) | Rotterdam criteria (2003) | National Natural Science Foundation of China (Grant No. 82004398) | No conflicts declared | Not mentioned |
| Nsugbe 2023 | Ejay Nsugbe | 2023 | England | Retrospective | Public database (Kaggle) | Not explicitly stated | Not mentioned | No conflicts declared | Not mentioned |
| Suha 2023 | Sayma Alam Suha | 2023 | Bangladesh | Retrospective | Public database (Kaggle) | Not explicitly stated. | None declared | No conflicts declared | Not mentioned |
| Lim 2023 | Jiekee Lim | 2023 | China | Prospective, Multicenter | Shanghai Municipal Hospital of Traditional Chinese Medicine, Shuguang Hospital and Yueyang Hospital; Hospital outpatient / clinical setting. | Chinese guidelines for diagnosis and treatment of polycystic ovary syndrome (2018) | Shanghai Key Laboratory of Health Identification and Assessment (21DZ2271000). | No conflicts declared | Not applicable (stated in article) |
| Lim 2023 | Jiekee Lim | 2023 | China | Retrospective, Multicenter . | Shanghai Municipal Hospital of Traditional Chinese Medicine and Shuguang Hospital; Hospital outpatient/clinical setting | Rotterdam criteria (2003) | Shanghai Key Laboratory of Health Identification and Assessment (21DZ2271000); Science and Technology Commission of Shanghai Municipality (21Y11923400). | No conflicts declared | Not mentioned |
| Sheikdavood 2023 | K. Sheikdavood | 2023 | India | Retrospective | Public database (Kaggle) | Not explicitly stated | Not mentioned | Not reported | Not mentioned |
| Elmannai 2023 | Hela Elmannai | 2023 | Saudi Arabia | Retrospective | Public database (Kaggle) | Not explicitly stated | Princess Nourah bint Abdulrahman University Researchers Supporting Project (PNURSP2023R330) | No conflicts declared | Not mentioned |
| Kumari 2023 | Ritika Kumari | 2023 | India | Retrospective | Public database (Kaggle) | Not explicitly stated | None declared | No conflicts declared | Not mentioned |
| Kumar 2024 | Ankur Kumar | 2024 | India | Retrospective | Public database (Kaggle) | Not explicitly stated | None declared | No conflicts declared | Not mentioned |
| Nethra 2024 | Nethra Sai M | 2024 | India | Retrospective | Not explicitly stated | Rotterdam criteria (2003) | Not mentioned | No conflicts declared | Not mentioned |
| Moral 2024 | Poonam Moral | 2024 | India | Retrospective | Public databases (Kaggle, PCOSGen, MMOTU) | Rotterdam criteria (2003) | DST under PURSE 2022 scheme | No conflicts declared | Not mentioned |
| Kermanshahchi 2024 | Jonathan Kermanshahchi | 2024 | America | Retrospective | Public database (Kaggle) | Not explicitly stated | Not mentioned | No conflicts declared | Not mentioned |
| Li 2024 | Nan Li | 2024 | China | Retrospective | Public databases ( GEO) | Not explicitly specified | Guangxi Medical high-level and sub backbone talents training "139" program (No. G202003005), the Key Research and Development Program of Guangxi (No. GuikeAB18126056) and Guangxi Self-Financing Research Program of Guangxi Region Health Commission (No. Z-B20221595, No. Z-B20221568). | No conflicts declared | Not mentioned |
| Rahman 2024 | Md Mahbubur Rahman | 2024 | Bangladesh | Retrospective | Public database (Kaggle) | Not explicitly stated | Not mentioned | No conflicts declared | Not mentioned |
| Sowmiya 2024. | Sowmiya S. | 2024 | India,Saudi Arabia | Retrospective, single-center | SRM Medical College Hospital and Research Centre | Ultrasound imaging (multiple follicles in ovary and irregular periods), confirmed by experienced sonologists | Researchers Supporting Project (RSPD2024R877), King Saud University, Saudi Arabia | No conflicts declared | Not mentioned |
| Rona 2024 | Günay Rona | 2024 | Turkey | Retrospective, single-center | Hospital database (inpatient/outpatient, from endocrinology department) | Rotterdam criteria (2003) | None declared | No conflicts declared | Not mentioned |
| Paramasivam 2024 | Gokila Brindha Paramasivam | 2024 | India | Retrospective | Public database (Kaggle) | Not explicitly stated | None declared | No conflicts declared | Not mentioned |
| Shamnugavadivel 2024 | Kogilavani Shanmugavadivel | 2024 | India,Saudi Arabia, Ethiopia | Retrospective | Public database (Kaggle) | Rotterdam criteria (2003) | Researchers Supporting Project (RSPD2024R846), King Saud University, Riyadh, Saudi Arabia | No conflicts declared | Not mentioned |
| Wang 2024 | Fengjuan Wang | 2024 | China | Prospective cross-sectional, single-center | Outpatient from Xijing Hospital | Rotterdam criteria (2003) | National Natural Science Foundation of China (No. 82300488, 81972026) and Shaanxi Provincial Key Research and Development Program | No conflicts declared | Not mentioned |
| Rajput 2024 | Ishwari Singh Rajput | 2024 | India | Retrospective | Public database (Kaggle) | Not explicitly stated; | Not mentioned | No conflicts declared | Not mentioned |
| Vairachilai 2024 | S. Vairachilai | 2024 | India | Retrospective | Public database (Kaggle ) | Not explicitly stated | None declared | No conflicts declared | Not mentioned |
| Umaa 2024 | Umaa Maheswari G | 2024 | India | Retrospective | Public database (Kaggle ), | Not explicitly stated; | Anna Research Fellowship scheme, Centre for Research, Anna University | No conflicts declared | Not mentioned |
| Ahmad 2024 | Rumman Ahmad | 2024 | India,Saudi Arabia | Retrospective | Public database (Kaggle) | Not explicitly stated | No external funding (stated in Funding section) | No conflicts declared | Not mentioned |
| Sumathi 2023 | M. Sumathi | 2023 | India | Retrospective, multi-center | Public database (Kaggle), images from Sathyabama General Hospital | Not explicitly stated | Department of Biotechnology, Government of India (Ref. No. AI-61/202(7) [BT/PR34231/AI/133/20/2019]) | No conflicts declared | Not mentioned |
| Tong 2025 | Cheng Tong | 2025 | China | Retrospective, Single-center | Outpatient clinic and Health Screening Centre of the First Affiliated Hospital of Zhejiang Chinese Medical University | Rotterdam criteria (2003) | None declared | No conflicts declared | Not mentioned |
| Smithakranthi2025 | A. Smithakranthi | 2025 | India | Retrospective | Public database (Kaggle) | Not explicitly stated | Not mentioned | No conflicts declared | Not mentioned |
| Oviya 2025 | Oviya Graselin S. | 2025 | India | Retrospective | Public database (Kaggle ) | Not explicitly stated | None declared | No conflicts declared | Not mentioned |
| Ghosh 2025 | Ananya Ghosh | 2025 | India | Retrospective | Public database (Kaggle) | Not explicitly stated | Vellore Institute of Technology (VIT), India | Not reported | Not mentioned |
| Faris 2025 | Najlaa Faris | 2025 | Iraq,Australia | Retrospective | Public database (Kaggle) | Not explicitly stated | Not mentioned | No conflicts declared | Not mentioned |
| Aldosary 2025 | Abdallah Aldosary | 2025 | Saudi Arabia | Retrospective, Multi-center | Dataset I: Hospital patients (India); Dataset II & III: Public gene expression databases (ArrayExpress) | Not explicitly stated | Prince Sattam bin Abdulaziz University (Project No. PSAU/2023/01/26692) | No conflicts declared | Not mentioned |
| Narni 2025 | Hanumanth Narni | 2025 | India | Retrospective, Case-Control, Single-center | Hospital (outpatient clinic based on context) | Ultrasonographic findings | None declared | No conflicts declared | Not mentioned |
| Panjwani 2025 | Bharti Panjwani | 2025 | India,Korea | Retrospective | Public databases (Kaggle) | Not explicitly stated | None declared | No conflicts declared | Not mentioned |
| Rona 2025 | Gunay Rona | 2025 | Turkey | Retrospective, Single-center | Hospital patients from Endocrinology Department, Kartal Dr. Lutfi Kirdar Training and Research Hospital | Rotterdam criteria (2003) | None declared | No conflicts declared | Not mentioned |
| Zad 2024 | Zahra Zad | 2024 | America | Retrospective, single-center | Outpatient electronic health records (EHR) from Boston Medical Center | A combination of ICD-9 code 256.4 and/or meeting the Rotterdam criteria | National Science Foundation (grants CCF-2200052, IIS-1914792, DMS-1664644), NIH (grants R01 GM135930, UL54 TR004130), Boston University Kilachand Fund for Integrated Life Science and Engineering | No conflicts declared | Not mentioned |
| Agirsoy 2025 | Mehtap Agirsoy | 2025 | America | Retrospective | Public database (Kaggle) | The 2023 International Evidence-Based Guideline, which refines the Rotterdam criteria | Not mentioned | No conflicts declared | Not mentioned |
| Jaganathan 2025 | Gowthami Jaganathan | 2025 | India | Retrospective | Public database (Kaggle) | Not explicitly stated | Not mentioned | No conflicts declared | Not mentioned |
| Yao 2025 | Bihua Yao | 2025 | China | Retrospective, multicenter | Hospital outpatient clinics from Department of Gynecology, The First People's Hospital of Jiashan and Jiaxing Hospital of Traditional Chinese Medicine | Rotterdam criteria (2003) | None reported | No conflicts declared | Not mentioned |
| Natarajan 2025 | Yuvaraj Natarajan | 2025 | India | Retrospective | Public database (Kaggle) | Not explicitly stated | Open access funding provided by Vellore Institute of Technology | No conflicts declared | Not mentioned |
| Sarwar 2025 | Muhammad Saood Sarwar | 2025 | Pakistan | Retrospective | Public database (Kaggle) | Not explicitly stated | Not mentioned | No conflicts declared | Not mentioned |
| Bharti 2025 | Bharti P. | 2025 | India | Retrospective | Public databases (Kaggle) | Rotterdam criteria (2003) | None reported | No conflicts declared | Not mentioned |
| Ramteke 2025 | Monali Ramteke | 2025 | India | Retrospective | Public databases (Kaggle) | Not explicitly stated | None reported | No conflicts declared | Not mentioned |
| Sundari 2025 | M. Shanmuga Sundari | 2025 | India | Retrospective | Public databases (Kaggle) | Rotterdam criteria (2003) | Not mentioned | No conflicts declared | Not mentioned |
| Pulluraparbil 2026 | Siji Jose Pulluraparbil | 2026 | India | Retrospective | Public databases (Kaggle) | Not explicitly stated | None reported | No conflicts declared | Not mentioned |
| Uddin 2026 | Khandaker Mohammad Mohi Uddin | 2026 | Bangladesh | Retrospective | Public database (Kaggle) | Rotterdam criteria (2003) | None reported | No conflicts declared | Not mentioned |
