# Supplementary material for: Machine learning and deep learning for diagnosis of Polyendocrine Metabolic Ovarian Syndrome: systematic review and meta-analysis
Source: Front Endocrinol (Lausanne). 2026 Jul 30;17:1889375. doi: 10.3389/fendo.2026.1889375 (PMC13468022; doi:10.3389/fendo.2026.1889375)
Supplement: Supplementary file 5 [file SupplementaryFile3.docx]

**Supplementary Data 3. The methodologies for model development and validation across the included studies**

| Study | Training Set (n) | Validation/Test Set (n) | Validation Type | Validation/Test Set Generation Method | Algorithm | Modeling Variables | Feature Selection Method | Model Calibration Metrics |
| --- | --- | --- | --- | --- | --- | --- | --- | --- |
| Cheng 2019 | 1000 labeled ultrasound reports | 1000 labeled ultrasound reports | Internal validation | Not explicitly stated | RBC, GBT model (using XGBoost). | Extracted ovarian volume; Specific n-grams indicating PCOM or volume confounders. | RBC, GBT | Not reported. |
| Lv 2022 | 576 subjects | 145 subjects | Internal validation | 5-fold cross-validation | CBAM, ResNet18, MIL | Scleral image pixels | CNNs | Not reported. |
| Zhang 2021 | 80 samples | 20 samples | Internal validation | Not explicitly stated | kNN, RF,XGB | Raman spectroscopy spectra | PCA | Not reported. |
| Xie 2022 | 30 | 30 | Internal validation | 10-fold cross-validation | LASSO, WGCNA, binary LR | NA methylation β-values at specific CpG sites (cg23464743, cg06834912, cg00103771) | WGCNA,LASSO | Not reported. |
| Sreejith 2022 | 80% of the dataset (after outlier removal) | 20% of the dataset (after outlier removal) | Internal validation | 10-fold cross validation | RDA, RF | Weight, BMI, Hb, Cycle length, FSH, LH, WHR, TSH, AMH, PRL, RBS, Weight Gain, Hair growth, Skin darkening, Hair loss, Follicle No. (L), Follicle No. (R), Average Follicle size (L), Average Follicle size (R), Endometrium (A mix of clinical, anthropometric, biochemical, and ultrasound features). | RDA | Not reported. |
| Xu 2022 | 8,204 cases | 3,516 cases | Internal validation | 10-fold cross validation | LASSO | ge, BMI, upper menstrual cycle limit (UML), AMH, testosterone (TES), androstenedione (AND), antral follicle count (AFC) | LASSO | Not reported. |
| Suha 2022 | 416 images | 178 images | Internal validation | k-fold cross-validation | VGGNet16, XGBoost | Ultrasound image pixels | CNN | Not reported. |
| Mehr 2022 | 487 | 54 | Internal validation | 10-fold cross-validation | Ensemble RF, Ensemble Extra Tree, AdaBoost, Multi-Layer Perceptron | 42 clinical and biochemical features | SBS, RF | Not reported. |
| Zigarelli 2022 | 421 | 105 | Internal validation | stratified K-fold cross-validation | CatBoost | Noninvasive (patient model): anthropomorphic (BMI, height, hip, waist, waist-hip ratio, weight), symptoms (acanthosis nigricans, acne, hair loss, hirsutism, irregular cycle, cycle length, weight gain), given (age, blood type, marriage years, abortion history, fast food, pregnancy, exercise) Invasive (provider model): above + test results (AMH, follicle counts/sizes, hormones, BP, glucose, etc.) | PCA | Not reported. |
| Silva 2021 | 131 | 15 | Internal validation | 10-fold cross-validation | BorutaShap, RF | Clinical/Demographics, Biochemical, Genetic, Ultrasound | BorutaShap | Not reported. |
| Tiwari 2022 | 378 | 162 | Internal validation | Hold-out validation (70% to 30%) | RF, SVM , LR, DT, KNN, Gradient Boosting, AdaBoost, XGBoost, CatBoost, LDA, QDA | Age, BMI, Cycle length, Follicle No. (L/R), Hormone levels (FSH, LH, AMH, PRL, TSH, Vitamin D3, Hb, etc.), Endometrium, Waist-Hip ratio, Skin/hair symptoms, etc. | Pearson’s correlation coefficient | Not reported. |
| Alamoudi 2023 | 312 images, 228 clinical cases | 78 images, 57 clinical cases | Internal validation | Hold-out validation (80% to 20%) | Image-only models: VGG16, VGG19, InceptionV3, DenseNet121, DenseNet201, MobileNet Fusion models: Joint Fusion Type I & II (combining image features with clinical data) | Image features: Ultrasound image pixels Clinical features: Age, marital status, BMI, weight, height, FSH, Testosterone, cycle regularity | Not reported | Not reported. |
| Wang 2023 | Not explicitly stated; used 80% of data in repeated sampling (10 times) | Not explicitly stated; used 20% of data in repeated sampling (10 times) | Internal validation | 10-fold cross-validation | MLP, XGBoost, SVM | Tongue parameters: TB-L, TB-a, TB-b, TC-L, TC-a, TC-b Pulse parameters: h3, h4/h1 Baseline: BMI | LASSO | Calibration curves presented; SVM model showed predicted probabilities closest to true probabilities. |
| Nsugbe 2023 | 80% of data used for training (exact number not specified) | 20% of data used for validation (exact number not specified) | Internal validation | 10-fold cross-validation (training set:test set=8:2) | DT, LDA, LR, KNN, SVM | Metabolic, physical, imaging, hormonal, biochemical. BMI, waist:hip ratio, and endometrium features | Not reported | Not reported. |
| Suha 2023 | 70% of data (exact number not specified after SMOTE). | 30% of data (exact number not specified after SMOTE). | Internal validation | Random split (70% to 30%) | LR, SVM, DT, KNN, NB, RF, GradBoost, XGBoost, AdaBoost, CatBoost, | Age, Weight, BMI, menstrual history (Cycle regularity, length), reproductive history (Pregnancies, abortions), symptoms (hair growth, skin darkening, pimples, weight gain), vital signs (BP, pulse), ultrasound measures (Follicle number/size in left/right ovary, Endometrium thickness), and biochemical markers (LH, FSH, AMH, TSH, PRG, Vit D3, Hb, RBS, etc.). | PCA,RFE | Not reported. |
| Lim 2023 | 75% of data (~344 subjects, exact number not specified) | 25% of data (~115 subjects, exact number not specified). | Internal validation | Stratified 10-fold cross-validation (training set:test set=75%:22%) | KNN, SVM, DT, RF, LR, SV, LSTM | 15 time-domain radial pulse wave parameters that were significantly different (p<0.01) between groups: right t1, and h4, t4, t, As, h4/h1, h3/h1, w/t from both left and right Guan (wrist) positions. | Univariate selection based on statistical significance (Wilcoxon rank sum test, p<0.05). | Not reported. |
| Lim 2023 | 315 subjects | 135 subjects | Internal validation | Stratified 10-fold cross-validation | ET, RF, XGBoost, SVM. | 12 Pulse Wave Parameters: left h1, h3, h5, t1, w/t; right h1, h4, h5/h1, t1, t5, As, Ad. 19 TCM Clinical Indices: BMI, menarche age, pregnancy times, slightly jelly-like menstrual texture, breast distension/pain, dysmenorrhea, cold aggravation, perimenstrual disorders, fatigue aggravation, abnormal leukorrhea, cold intolerance, spontaneous sweating, lassitude, bad breath, dry mouth, sweet food preference, sleep disorder, negative emotions, family history of hypertension, delayed menstruation, postgraduate education, TCM tongue signs (dark red, dark, spotted, greasy coating). | XGB, RF, ET | Not reported. |
| Sheikdavood 2023 | Not explicitly split; entire dataset of 541 records used with cross-validation. | Not explicitly defined; evaluated via k-fold cross-validation. | Internal validation | k-fold cross-validation（k=10,20,30,40） | IAKmeans-RSA, CNN, DNN | Clinical: BMI, weight, waist, hip, cycle regularity, skin darkening, hair growth, weight gain, pimples, hair loss, fast food consumption. Ultrasound: Follicle number (left/right), average follicle size. Biochemical: AMH, LH, FSH. | CNN | Not reported. |
| Elmannai 2023 | Not explicitly stated; data split into training sets using 70:30 and 80:20 ratios. | Not explicitly stated; corresponding test sets were 30% and 20% of the data, respectively. | Internal validation | Stratified 10-fold cross-validation ( 80% to 20%, 70% to 30) | LR, RF, DT, NB, SVM, KNN, XGBoost, AdaBoost, Stacking ML (with RF as meta-learner) | Clinical/Demographic: Age, Weight, Height, BMI, Blood Group, Pulse Rate, Respiration Rate, Hemoglobin, Cycle length, Marriage status, Pregnant status, No. of abortions, Weight gain, Hair growth, Skin darkening, Hair loss, Pimples, Fast food, Regular exercise, Blood pressure (systolic/diastolic). Ultrasound: Follicle No. (left/right). Biochemical: FSH, LH, FSH/LH ratio, TSH, AMH, Prolactin (PRL), Vitamin D (Vit_D3), Progesterone (PRG), Random Blood Sugar (RBS), Beta-HCG | RFE, RF | Not reported. |
| Kumari 2023 | 379 instances | 162 instances | Internal validation | Stratified 10-fold cross-validation | LR, SVM, DT, RF, NB, AdaB, Stack-LR, Stack-SVM, Stack-DT, Stack-RF, Stack-NB, Stack-AdaB | Clinical/Demographic: Age, Weight, Height, BMI, Blood Group, Pulse rate, Respiration rate, Hemoglobin, Cycle regularity, Cycle length, Marriage status, Pregnant status, No. of abortions, Weight gain, Hair growth, Skin darkening, Hair loss, Pimples, Fast food, Regular exercise, Blood pressure (systolic/diastolic). Ultrasound: Follicle No. (left/right), Avg. follicle size (left/right), Endometrium thickness. Biochemical: FSH, LH, FSH/LH ratio, TSH, AMH, Prolactin (PRL), Vitamin D3, Progesterone (PRG), Random Blood Sugar (RBS), Beta-HCG. | Backward Feature Elimination (BFE) | Not reported. |
| Kumar 2024 | 70:30 split: ~379 instances (70% of 541).  80:20 split: ~433 instances (80% of 541) | 70:30 split: ~162 instances.  80:20 split: ~108 instances. | Internal validation | Stratified 10-fold Cross Validation ( 80% to 20%, 70% to 30) | LR, LDA, GNB, SVM, XGBoost, DT, AdaBoost , RF, KNN, All models were hybridized with Particle Swarm Optimization (PSO) | Clinical/Demographic: Age, Weight, Height, BMI, Blood Group, Pulse Rate, Respiration Rate (RR), Hemoglobin (Hb), Cycle regularity/length, Marriage Status, Pregnant status, No. of abortions, Weight gain, Hair growth, Skin darkening, Hair loss, Pimples, Fast food, Regular exercise, Blood pressure (Systolic/Diastolic). Biochemical: FSH, LH, FSH/LH ratio, I/II beta-HCG, TSH, AMH, Prolactin (PRL), Vitamin D3 (Vit D3), Progesterone (PRG), Random Blood Sugar (RBS). Ultrasound: Follicle No. (L/R), Avg. Follicle size (L/R), Endometrium thickness. Anthropometric: Hip, Waist, Waist:Hip Ratio. | correlation heatmaps (Pearson) and Exploratory Data Analysis (EDA) | Not reported. |
| Nethra 2024 | 3,200 ultrasound images | 1,468 ultrasound images | Internal validation | hold-out validation | ResNet-50, SVM, RF, LR, DT | Clinical: Age, Weight, Height, BMI, Cycle length, Weight gain, Skin darkening, Hair growth, Hair loss, Pimples, Fast food, etc. Ultrasound: Extracted features via ResNet-50 (e.g., cyst patterns, ovarian morphology) | Pearson correlation coefficient | Not reported. |
| Moral 2024 | 70% of Kaggle dataset (1,347 images) | 30% of Kaggle dataset (577 images) | External validation | 5-fold cross-validation | CystNet, RF, AdaBoost, K-NN, NB | Extracted image features (2,048 from InceptionNet V3 + 1,024 from Autoencoder = 3,062 features total) | Not reported | Not reported. |
| Kermanshahchi 2024 | 80% of images (1,546 images) | 10% validation (193 images), 10% testing (193 images) | External validation | Random split (80%, 10%, 10%) | CNN | Ultrasound image features | Not reported | Not reported. |
| Li 2024 | 28 samples (15 PCOS, 13 normal). | not provided | External validation | 10-fold cross-validation | RFE, LASSO COX regression, WGCNA, ssGSEA | MMP25, CSF3R, SLPI, MMP9, CLEC4E, and SIGLEC10. | RFE, LASSO COX,WGCNA | Not reported. |
| Rahman 2024 | 80% of data (432 patients) | 20% of data (109 patients) | Internal validation | Random split (80%, 20%) | LR, DT, AdaBoost, RF, SVM, KNN, Gaussian NB, Bernoulli NB, Multinomial NB, Gradient Boosting, MLP, Extra Tree, Linear SVC | clinical, biochemical, ultrasound, lifestyle | Not reported | Not reported |
| Sowmiya 2024. | 80% of each dataset | 20% of each dataset | Internal validation | Random split (80%, 20%) | k-Star, SGD, RF, MobileNet, ResNet152V2, DenseNet121, Vision Transformer, Custom F-Net | contrast, energy, homogeneity, correlation, entropy, skewness, kurtosis, mean, variance, standard deviation | Not reported | Not reported |
| Rona 2024 | 141 ovaries | 61 ovaries | Internal validation | 10-fold cross-validation | RF, GB, Light GBM, NB, KNN, Extra Trees, LR | Radiomic features from T2-weighted MRI (first-order, shape, texture, wavelet-based) | LR, LASSO | Not reported |
| Paramasivam 2024 | 224 images | 28 images (testing) and 25 images (validation) | Internal validation | Random split | Self-Defined CNN,SVM, RF, LR | Image features extracted from CNN layers | CNN | Not reported |
| Shamnugavadivel 2024 | Total Training Set Size (Clinical): 433 patients; Total Training Set Size (Ultrasound): 1924 images | Total Validation/Test Set Size (Clinical): 108 patients; Total Validation/Test Set Size (Ultrasound): 1932 images | Internal validation | Not explicitly stated | Clinical data:LR, NB, SVM  Ultrasound images: CNN, VGG16 | age, weight, height, BMI, testosterone, menstrual disorder, hair growth, skin darkening, follicle count, hormone levels (LH, FSH, TSH, AMH, PRL, etc.), and lifestyle factors (fast food). | Pearson correlation | Not reported |
| Wang 2024 | Not explicitly split; 5-fold cross-validation used | Not explicitly defined; cross-validation used | Internal validation | 5-fold cross-validation | AdaBoost, XGBoost, DT, KNN, LR, RF | LH/FSH, Prog, TC, TG, HDL-C, AMH, OVGP1 (added later) | Recursive Feature Elimination with Cross-Validation (RFE-CV) | Not reported |
| Rajput 2024 | Varies by split ratio (70%, 80%, 90% of data) | Corresponding 30%, 20%, 10% of data | Internal validation | Train-Test Split | RF, DT, NB, Neural Network, XGBoost | age, BMI, cycle regularity, hormone levels, follicle count, endometrial thickness, symptoms | Sine Cosine Algorithm (SCA) compared with FeatureWiz, PCA, PSO, GA, NSGA-II | Not reported |
| Vairachilai 2024 | Not explicitly stated; dataset split into training and test sets | Not explicitly stated | Internal validation | Not explicitly stated | Traditional ML: LR, SVM, KNN, DT, NB.  Ensemble Methods: RF, XGBoost, AdaBoost, CatBoost, Blending. | ollicle No. (R), Follicle No. (L), Skin darkening, Hair growth, Weight gain, Cycle (R/I), Fast food, Pimples, AMH (ng/mL), Weight (Kg), BMI, Hair loss, Waist (inch), Hip (inch), Avg. F size (L) (mm), Endometrium (mm). | Pearson correlation coefficient | Not reported |
| Umaa 2024 | Not explicitly stated; used 80:20 and 70:30 splits for tuning | Not explicitly stated; used 80:20 and 70:30 splits for tuning | Internal validation | 8-fold cross-validation | LR, SVM, DT, RF, NB, KNN, AdaBoost, XGBoost, Extra Trees Stacking Ensemble models (2 types) | Clinical: Age, BMI, Weight, Height, Waist, Hip, Waist-Hip Ratio, BP, Pulse, RR, etc. Biochemical: Hb, FSH, LH, AMH, TSH, PRL, Vit D3, RBS, etc. Ultrasound: Follicle No. (L/R), Avg. Follicle Size (L/R), Endometrium thickness Lifestyle: Fast food, Regular exercise, Weight gain, etc. Symptoms: Hair growth, Skin darkening, Hair loss, Pimples | Threshold-driven Optimized PCA (TOPCA) , Optimized Salp Swarm Method (OSSM) – , Threshold-driven Optimized Mutual Information Method (TOMM) | Not reported. |
| Ahmad 2024 | 540 samples (after SMOTE) | 88 samples | Internal validation | Not explicitly stated (80%, 20%) | SMOTE + LSTM SMOTE + 1D CNN SMOTE + CNN-LSTM (stacked) | Clinical: Age, Weight, Height, BMI, BP, Pulse, RR Biochemical: Hb, FSH, LH, AMH, TSH, PRL, RBS, Vit D3, etc. Ultrasound: Follicle No. (L/R), Avg. Follicle Size (L/R), Endometrium thickness Symptoms & Lifestyle: Hair growth, Skin darkening, Weight gain, Fast food, Exercise | Pearson correlation coefficient | Not reported. |
| Sumathi 2023 | Not explicitly stated | Not explicitly stated | Internal validation | Not explicitly stated | DarkNet-19, AlexNet, SqueezeNet, SVM | Image-based features | Not used; all extracted features employed | Not reported |
| Tong 2025 | 139 participants | 20 participants | Internal validation | 10-fold cross-validation | XG Boost, RF, SVM, LR, Gaussian Naive Bayes | LH, LH/FSH, E2, PRL, T, AMH, AD (Androstenedione), COR (Corticosterone). | LASSO | Not reported |
| Smithakranthi2025 | Not explicitly stated as a single number. The study used split ratios (80/20 and 70/30) on the total dataset (n=541) | Not explicitly stated as a single number (depends on the split ratio used: e.g., 20% of 541 ~108 participants) | Internal validation | Not explicitly stated (80%, 20%; 70%, 30%) | MobileNetV3 (pre-trained CNN), 1D-CNN, LSTM. SMOTE was used for data balancing. | Follicle count (right ovary - FL_R) and Follicle count (left ovary - FN_L). | Mutual Information (MI) method was used for feature selection from the 43 original variables. | Not reported |
| Oviya 2025 | 70% of 541 instances (379). | 30% of 541 instances (162). | Internal validation | Not explicitly stated (70%, 30%) | ATSA , XGBoost | Weight, HB, AMH, Cycle length, FSH, BMI, LH, PRL, WHR, RBS, TSH, Hair growth, Skin darkening, Hair loss, Endometrium thickness, and Number/Average follicles in left and right ovaries. | Enhanced Tunicate Search Algorithm (ETSA/ATSA) | Not reported |
| Ghosh 2025 | 5600 images (after augmentation: 4000 PCOS and 4000 Non-PCOS, 70% train split) | 2400 images (30% test split) | Internal validation | 10-fold cross-validation | Ensemble of EfficientNetB7 and DenseNet201 (transfer learning) | Ultrasound image pixel data | Convolutional layers of pre-trained models | Not reported |
| Faris 2025 | 70% of 538 samples (after preprocessing) | 30% of 538 samples | Internal validation | 10-fold cross-validation | Random Subspace with Bootstrap Aggregating Ensembles (RSBE) – ensemble of Linear SVM, Gaussian SVM, KNN, DT, NB | Age, BMI, Cycle length, Follicle number, Prolactin, LH, FSH, TSH, AMH, Progesterone, hair growth, pimples, etc. | Combined K-means clustering and Genetic Algorithm (GA) | Not reported |
| Aldosary 2025 | Not explicitly split | Not explicitly reported | Internal validation | Not explicitly stated | SLOGPO: Stacked Ensemble Learning + Improved Giza Pyramids Optimizer (IGPO) PCOSNet: 1D Convolutional Neural Network (1D-CNN) | Clinical/Physical (Dataset I): Age, Weight, Height, BMI, Cycle length, AMH, LH, FSH, Follicle No., etc. (42 features total) Genetic (Dataset II): 54,675 gene features (skeletal muscle) Genetic (Dataset III): 10 gene features (cumulus cells) | BORUTA, Genetic Algorithm (GA), Recursive Feature Elimination (RFE), PCA | Not reported |
| Narni 2025 | 160 (80% of 200) | 40 (20% of 200) | Internal validation | 5-fold cross-validation | DT, NB | Clinical/Lifestyle: Abnormal BMI, Abnormal WHR, Physical Inactivity, Weight Gain, Hair Loss, Hirsutism, Irregular Cycles, Stress, Skin Darkening, Fast-Food Consumption. | Chi-square filter method | Not reported. |
| Panjwani 2025 | 720 samples (80% of 900) | 180 samples (20% of 900) | Internal validation | 10-fold cross-validation | Base Learners:KNN, SVM, LR, DT, RF, XGB, - MLP. Ensemble Model (EL): Stacking ensemble of the 7 base learners. Meta-classifier: MLP. Optimized Models: RSOEL, CSOEL, WaOEL. | Resting blood pressure (restbps), maximum heart rate (thalach), cholesterol (chol), chest pain type (cp), resting electrocardiogram (restecg), fasting blood sugar (fbs), random glucose (glucose), body mass index (BMI), weight gain (Y/N), waist-to-hip ratio, cycle length, hair growth (Y/N). | Features were selected based on domain knowledge during dataset creation: relevant attributes related to hypertension, diabetes, obesity, and cardiometabolic disease were retained from the source datasets, while complex clinical test parameters were discarded. | Not reported |
| Rona 2025 | 196 ovaries | 84 ovaries | Internal validation | 10-fold cross-validation | LightGBM, K-Neighbors Classifier, Extra Trees Classifier, RF Classifier, Ridge Classifier, LDA, LR, Gradient Boosting Classifier, Ada Boost Classifier, NB, SVM , DT, Quadratic Discriminant Analysis, Dummy Classifier. | Imaging (Radiomics) | RF, LASSO,LR | Not reported |
| Zad 2024 | The dataset was split into 5 random parts, with 4 parts used for training (80%) | One part was used for testing (20%) | Internal validation | 5-fold cross-validation | LR, SVM, GBT/GBM, RF | Demographics/Anthropometrics: age, race, smoking, marital status, homelessness, education, BMI categories. Cardiovascular: hypertension category;Reproductive/Endocrine: bHCG status, gravidity, MLP score, TSH, A1c, LDL, HDL, hypercholesterolemia; Others: HIV status, age at menarche, pelvic inflammatory disease, hysterosalpingogram history. | Recursive Feature Elimination (RFE) | Not reported |
| Agirsoy 2025 | 433 | 108 | Internal validation | Random splitting | ANN, SVM, LR, KNN, XGBoost | Clinical: age, BMI, cycle length, hair growth (hirsutism), skin darkening, weight gain, hair loss, pimples, fast food consumption; Biochemical: FSH, LH, AMH, total testosterone, free testosterone, TSH, Vit D3; Ultrasound: Follicle number, Average follicle size, Endometrium thickness. | XGBoost, SHAP | Not reported |
| Jaganathan 2025 | 541 | 162 | Internal validation | Random split | An ensemble of RF and XGBoost | BMI, follicle number, age, weight, height, pulse rate, etc | Kendall correlation method | Not reported |
| Yao 2025 | 1,120 | 283 | External validation | Random split | LGBM, RF, SVM, XGBoost, DT, KNN, LR | Age, Androstenedione, DHT, 17α-OHP, Estrone, LH, Progesterone, Testosterone, FSH, Estradiol, Prolactin, DHEAS, AMH, INS-0h, INS-0.5h, INS-1h, INS-2h, INS-3h, Total Cholesterol, Triglycerides, ApoE, Lp(a), Glucose | LASSO | Calibration curves showed good fit. Brier scores: Train cohort 0.129, Test cohort 0.140. |
| Natarajan 2025 | 45,600 images | 760 original images (360 for testing and 400 for validation after split). | Internal validation | Random split | FCAU-Net (Feature Fusion Context Attention U-Net) | Ultrasound image pixels | Not reported | Not provided |
| Sarwar 2025 | 1,924 images | 1,922 images | Internal validation | Original dataset split (predefined training and test folders). | QCNN, RF, SVM, GB, XGBoost, LightGBM, CatBoost, MLP. | Ultrasound image pixels | Not reported | Not reported. |
| Bharti 2025 | 720 samples | 180 samples | Internal validation | Random split | KNN, LR, SVM, DT, RF, XGB, DL, Ensemble Learning (stacking) | Resting blood pressure, max heart rate, cholesterol, chest pain type, resting ECG, fasting blood sugar, random glucose, BMI, weight gain, waist-to-hip ratio, cycle length, hair growth | Not reported | Not reported |
| Ramteke 2025 | 433 samples | 108 samples | Internal validation | Random split | RF, DT, LGBM, CatBoost, AdaBoost, XGBoost, ExtraTrees, Soft Voting Classifier | Follicle No R, Fast food, Cycle, Weight gain, Age, BMI, Follicle No R, Follicle No L, AMH, FSH, LH, etc. | Sequential Forward Selection (SFS) and Boruta | Not reported |
| Sundari 2025 | Not explicitly stated | Not explicitly stated | Internal validation | Random split (80:20) | Enhanced EfficientNet-B3 | Image Features; Clinical Biomarkers: Serum LH, FSH, AMH | Not applicable (deep learning feature extraction) | Not reported |
| Pulluraparbil 2025 | 509 | validation (145), testing (73) | Internal validation | Random split for Kaggle/UCI datasets; external datasets used for independent testing | SMOTE-ENN, GS2TM, DASCD | Age, Weight, BMI, Blood Group, Weight gain (Y/N), Hair growth (Y/N), Fast food (Y/N), Regular Exercise (Y/N), Follicle No. (L/R), Avg. Follicle Size (L), Endometrium thickness, AMH, PRL, PRG, RBS, Pregnant (Y/N), No. of abortions, FSH, FSH/LH ratio, Hip, Waist, Waist:Hip Ratio | Gorilla Salp Swarm Troop Model (GS2TM) | Not reported |
| Uddin 2026 | 432 (80% of 541 after an 80:20 split, before SMOTE application on the training portion). Post-SMOTE, the balanced training set had 584 instances (292 PCOS, 292 non-PCOS). | 109 | Internal Validation | Random split | MLP, CatBoost classifiers | The final model used the top 10 selected features: Follicle No. (R), Follicle No. (L), Weight gain (Y/N), Cycle (R/I), Skin darkening (Y/N), hair growth (Y/N), Cycle length(days), Fast food (Y/N), Waist:Hip Ratio, LH(mIU/mL). | Mutual Information and Extra Trees Classifier | Not reported |
