# Supplementary material for: Machine learning and deep learning for diagnosis of Polyendocrine Metabolic Ovarian Syndrome: systematic review and meta-analysis
Source: Front Endocrinol (Lausanne). 2026 Jul 30;17:1889375. doi: 10.3389/fendo.2026.1889375 (PMC13468022; doi:10.3389/fendo.2026.1889375)
Supplement: Supplementary file 6 [file SupplementaryFile4.docx]

**Supplementary Data 4. Methodological Quality and Reporting of included studies**

| **Study** | **Blinding in gold standard** | **TRIPOD-ML/STARD guidelines** | **Code and data accessibility** | **Key limitations discussed by the authors** |
| --- | --- | --- | --- | --- |
| Cheng 2019 | Not reported. | Not reported. | Code: Not mentioned. Data: Available from the corresponding author on reasonable request. | (1) The method infers PCOM status from radiology report text, not directly from ultrasound images. It is a proxy measure. (2) Radiologists may not describe all relevant findings in the report (e.g., follicle count may be omitted), so absence of mention in text does not guarantee absence in the image. (3) The RBC had difficulty with temporal language (e.g., past vs. current cysts). (4) Both models had errors when ovarian volume could not be reliably extracted (e.g., due to typographical errors). (5) The GBT model, not using explicit sentence splitting, could misinterpret long-range word interactions (e.g., associating a cyst with the wrong ovary). |
| Lv 2022 | Not reported. | Not reported. | Code: Not mentioned. Data: "The raw data supporting the conclusions of this article will be made available by the authors, without undue reservation." | (1) Dataset is not large enough and lacks external data for evaluating generalization ability. (2) Heterogeneity of PCOS necessitates a larger, more diverse dataset (wider range of groups). (3) Data masking: Only PCOS status was available; effects of specific medical conditions, race, and geography were not considered. (4) Limited interpretability: Some visualization results (Grad-CAM) are difficult to explain pathologically. More physiological study is needed to link scleral changes to PCOS. |
| Zhang 2021 | Not reported. | Not reported. | Code and Data: https://1drv.ms/u/s!AueePmluaEFYG_VJW_zMi7JkWVV6lg | (1) Small sample size (n=100). (2) Follicular fluid samples were obtained under controlled ovarian hyperstimulation (not natural cycles), which may affect metabolic profiles. (3) Collection of follicular fluid is invasive, limiting its clinical utility, especially for adolescents or women not seeking fertility treatment. (4) The model performance was lower for plasma samples compared to follicular fluid. |
| Xie 2022 | Not reported. | Not reported. | Code: Not mentioned. Data: Publicly available from the GEO database (GSE80468). | (1) The identified biomarkers were based on retrospective data; prospective clinical validation is needed. (2) The causal correlation between the alterations in DNA methylation and the development of PCOS was not investigated experimentally. |
| Sreejith 2022 | Not reported. | Not reported. | Code: Not mentioned. Data: The dataset is publicly available on Kaggle. | (1) The study was limited to combining the Red Deer Algorithm with the Random Forest classifier. Testing other classifiers (e.g., Artificial Neural Networks) optimized with RDA might yield different results. (2) The study did not test the proposed framework on other clinical datasets to establish its generalizability/scalability. |
| Xu 2022 | Not reported. | Not reported. | Original data included in article/Supplementary Material; further inquiries to corresponding author | (1) Retrospective design; need for prospective validation.(2) Variability in AMH assays across manufacturers. (3) Poor accuracy/sensitivity of testosterone assays in women. (4) Need for additional predictors to improve model performance. (5)Model not suitable for adolescents (only reproductive-aged women from infertility clinics) |
| Suha 2022 | Not reported. | Not reported. | Available from corresponding author on reasonable request (not publicly available) | (1 ) Limited dataset size (594 images). (2) Model is a “black box” with poor explainability. (3) Lack of external validation. (4) Images collected from varied sources may have quality variability. (5) No clinical/demographic data integrated. |
| Mehr 2022 | Not reported. | Not reported. | Original dataset: publicly available on Kaggle Processed dataset and code: available upon request | (1) Dataset from a single geographical region (India), limiting generalizability. (2) Need for clinical expert validation of selected features. (3)Feature selection may ignore clinically important variables if overly aggressive. |
| Zigarelli 2022 | Not reported. | Not reported. | Data: publicly available on Kaggle Code: not specified. | (1) Dataset limited to a specific population (Kerala, India). (2) Possible heterogeneity in diagnostic criteria across hospitals. (3) Lack of psychological variables in the model. (4) Need for larger multi-ethnic datasets for generalization. |
| Silva 2021 | Not reported. | Not reported. | Code: The analysis used Python and scikit-learn. No link to a specific repository is provided, but the general library is cited. Data: "The datasets generated during the current study are not publicly available but are available from the corresponding author on reasonable request." | (1) Difficulty in clinically distinguishing PCOS from nonclassic adrenal hyperplasia based on hyperandrogenism alone. (2) Relatively small sample size and cross-sectional design. (3) Need for external validation in other populations (same region and different ethnic backgrounds) to assess generalizability. (4) The unexpected finding of lower CRP in the PCOS group within the SHAP model needs replication. |
| Tiwari 2022 | Not reported. | Not reported. | Data: Publicly available on Kaggle (Kottarathil, 2020) Code: Not mentioned | (1) Performance dependent on dataset nature, sampling, and preprocessing. (2) Lack of multi-modality data (e.g., ultrasound images). (3) Single dataset used; suggests future use of larger/more diverse datasets. |
| Alamoudi 2023 | Not reported. | Not reported. | Code: Available upon request Data: Restricted due to patient privacy (Ethics Committee of Imam Abdulrahman bin Faisal University) | (1) Limited sample size. (2) Single-center data. (3) Lack of computational resources. (4) Only one ultrasound image per patient used. (5) Clinical dataset could be improved with more symptoms (e.g., acne, hirsutism) |
| Wang 2023 | Not reported. | Not reported. | Code: Not mentioned Data: Available from corresponding author upon reasonable request | (1) Limited sample size. (2) Single-center data. (3) Tongue and pulse data collection may be influenced by operator subjectivity. (4) Model generalizability needs further validation with larger, multi-center datasets. |
| Nsugbe 2023 | Not reported. | Not reported. | Data publicly available (Kaggle). Code availability not mentioned. | Limited sample size and homogeneity; need for more robust and varied dataset for clinical validation; future work suggested on regression-based learners for continuous staging and feature selection for parsimony. |
| Suha 2023 | Not reported. | Not reported. | Data is publicly available (Kaggle). Code availability is not mentioned. | 1) Use of a single, limited-size dataset from an open source (lack of large, real-time data). 2) Choice of base classifiers was based on prominence in literature; performance might differ with other classifiers. 3) The numbers of reduced features (35,30,25,20) were chosen arbitrarily. 4) Lack of explainable AI (XAI) components to justify predictions. |
| Lim 2023 | Not reported. | TRIPOD guidelines were followed | Data is not publicly available due to ethical concerns but available from the corresponding author upon reasonable request. Code availability not mentioned. | 1) Potential noise/bias in pulse signal collection (patient tremors, respiration, instrument vibration). 2) Technological limitations in fully distinguishing noise from pulse signals. 3) Relatively small and imbalanced sample size (70% PCOS vs. 30% healthy). 4) Analysis limited to time-domain parameters only. |
| Lim 2023 | Not reported. | TRIPOD guidelines were followed | Data is not publicly available due to patient privacy but can be requested from the corresponding author for qualified researchers. Code availability not mentioned. | Small sample size. |
| Sheikdavood 2023 | Not reported. | Not reported. | Data: Publicly available via Kaggle Code: Not specified (likely not provided) | (1) Small dataset size (541 records); (2) Future work needed with larger datasets and optimization algorithms to reduce complexity. |
| Elmannai 2023 | Not reported. | Not reported. | Data: Publicly available on Kaggle Code: Not specified (likely not provided) | (1) Dataset limitations: missing values, outliers, class imbalance.(2) Generalizability: model performance on larger, more diverse datasets unknown. (3) Potential overfitting due to small dataset size.(4) Need for external validation in clinical settings. |
| Kumari 2023 | Not reported. | Not reported. | Data: Publicly available on Kaggle Code: Not specified (likely not provided) | (1) Did not evaluate the model with different feature selection methods. (2) Need for further testing with more datasets and classifiers. |
| Kumar 2024 | Not reported. | Not reported. | Data: Publicly available on Kaggle. Code: Not specified (likely not provided). | The need for validation of the model on larger and more diverse datasets to assess generalizability. |
| Nethra 2024 | Not reported. | Not reported. | Not reported | Use of a limited dataset size  (1) Need for more diverse and larger datasets. (2) Lack of incorporation of additional etiological factors. (3) Need for improved methods to predict hyperandrogenism with more features. (4) Potential for generalization issues due to single-center data. |
| Moral 2024 | Not reported. | Not reported. | Data publicly available via Kaggle and other repositories; code availability not stated | (1) Dataset from single source may not represent population diversity. (2) Computational demands of CystNet may limit deployment on low-resource devices. (3) Need for multi-source datasets to improve generalizability. (4) Challenges in real-time clinical integration and user-friendly interface development. |
| Kermanshahchi 2024 | Not reported. | Not reported. | Data publicly available via Kaggle; code availability not stated | (1) Small dataset size. (2) Unreliable image quality. (3) Model possibly overfitted. (4) Only ultrasound images used, missing other diagnostic criteria (clinical exam, lab values). (5) Lack of generalizability due to limited dataset. |
| Li 2024 | Not reported. | Not reported. | Data are publicly available in the GEO repository. Code availability is not stated. | (1) The study is based on bioinformatics analysis; further in vitro and in vivo experiments are needed to validate the functions of the identified hub genes. (2)The specific regulatory mechanisms of the newly identified genes (CLEC4E, SIGLEC10) in PCOS require further investigation. (3) The sample size, while derived from multiple datasets, may still be limited. (4) The study focuses on autophagy-related gene expression and does not incorporate other clinical diagnostic modalities (e.g., imaging, physical exam). |
| Rahman 2024 | Not reported. | Not reported. | Data available on GitHub (https://github.com/shimulmbstu/PCOS); code availability not explicitly stated | Not explicitly listed |
| Sowmiya 2024. | Not reported. | Not reported. | Data: Available on GitHub (https://github.com/Sowmiya-1999/Sowmiya) Code: Not specified | (1) Lack of clinical validation in real-world settings. (2)Black-box nature of DL models. (3) Scalability and integration challenges in clinical workflows. (4) Data privacy and regulatory compliance issues. (5) Need for frequent model updates with new data |
| Rona 2024 | Not reported. | Not reported. | Not publicly available. | (1) Retrospective design. (2) Small sample size. (3) Did not consider PCOM appearance on ultrasound in phenotype grouping. (4) Single-center study. |
| Paramasivam 2024 | Not reported. | Not reported. | Data partially from Kaggle; code availability not stated | (1) Small dataset size. (2) Difficulty in obtaining medical images. (3) Lack of clinical feature integration (e.g., diet, lifestyle, symptoms like hair loss, weight gain). (4) Need for validation with data from diverse hospitals before clinical deployment |
| Shamnugavadivel 2024 | Not reported. | Not reported. | Data available on Kaggle; code availability not stated | Not explicitly listed in a limitations section, but the discussion highlights challenges in PCOS diagnosis (symptom variability, lack of single test, manual follicle counting). No specific limitations of the study methodology are detailed. |
| Wang 2024 | Not reported. | Not reported. | Data available in article/Supporting Information; code not mentioned | (1) Small sample size. (2) Single-center design, need for multicenter validation. (3) Some control participants had irregular menstruation. (4) Lack of multilayer distribution analysis. (5) Need for functional studies (cell/animal experiments) to explore OVGP1 mechanism |
| Rajput 2024 | Not reported. | Not reported. | Data available on Kaggle; code not mentioned (available on request per data availability statement) | (1) Model evaluated on only one dataset. (2) Lack of external validation on diverse populations. (3) Data security and patient privacy concerns. (4) Need for cloud-based deployment for broader validation. |
| Vairachilai 2024 | Not reported. | Not reported. | Data available on Kaggle; code not mentioned | (1) Dataset from a single region (Kerala, India) may limit generalizability. (2) Use of SMOTE may introduce artificial variability. (3) Model complexity could affect interpretability. (4) Lack of validation in real-world clinical settings. |
| Umaa 2024 | Not reported. | Not reported. | Data: Publicly available on Kaggle  Code: Not specified | (1) Limited dataset size and scope may reduce generalizability. (2) Potential biases in model integration with stacking ensembles; (3) Compatibility issues in building the smart predictor with stacking approaches. (4) Need for larger and more diverse datasets, including genetic and longitudinal data. (5) Requires further validation in clinical settings. |
| Ahmad 2024 | Not reported. | Not reported. | Data: Publicly available on Kaggle  Code: Not specified | (1) Small dataset size may limit generalizability. (2) Risk of overfitting due to limited samples. (3) Lack of external validation on independent datasets. (4) Use of synthetic data (SMOTE) may introduce bias. |
| Sumathi 2023 | Not reported. | Not reported. | Data: Publicly available via Kaggle and Google Drive link Code: Not specified | (1) Limited dataset size; need for larger real-time database to improve accuracy. (2) No clinical/demographic data integrated; only image-based features used. (3) Lack of external validation |
| Tong 2025 | Not reported. | Not reported. | Data is stated to be "available from the corresponding author on reasonable request." Code availability is not mentioned. | (1) Small sample size (total n=159, validation n=20) and single-center design, leading to potential selection bias and limited generalizability. (2) Lack of an external validation dataset. (3) Key metabolic indicators (e.g., insulin resistance parameters like HOMA-IR) were not included. (4) Some potentially valuable information (e.g., detailed family history, waist-to-hip ratio) was missing. (5) Obesity was not a prominent feature in their cohort, possibly due to population-specific habits or small sample size. |
| Smithakranthi2025 | Not reported. | Not reported. | The dataset is publicly available on Kaggle. Code availability is not mentioned. | (1) Small dataset size (n=541). (2) Class imbalance (177 PCOS vs. 364 non-PCOS). (3) Risk of overfitting due to dataset size, which was addressed using SMOTE and early stopping. (4) Need for validation on larger, independent datasets. |
| Oviya 2025 | Not reported. | Not reported. | Data is stated to be "made available upon reasonable request." Code availability is not mentioned. | (1) Dependence on large, high-quality datasets; risk of overfitting with limited or biased data. (2) Heterogeneity of PCOS presentation makes developing a universal model challenging. (3) The "black box" nature of machine learning models, which hinders interpretability. (4) Limited generalizability to different populations due to demographic or healthcare practice differences. (5) Potential oversight of important clinical context (patient history, comorbidities). (6) Complex regulatory landscape for medical machine learning applications. |
| Ghosh 2025 | Not reported. | Not reported. | Data: Publicly available on Kaggle Code: Not mentioned (likely not provided) | (1) Dependence on high-quality ultrasound images. (2) Computational complexity due to ensemble and GA. (3) GA-based tuning is computationally intensive. (4) Generalization to diverse populations not fully validated |
| Faris 2025 | Not reported. | Not reported. | Data: Publicly available on Kaggle Code: Not mentioned (likely not provided) | (1) Dependence on data quality (missing values, noise). (2) Need for larger and more diverse datasets for generalization. (3) Computational cost of ensemble and GA-based feature selection. (4) Comparison with deep learning methods limited by data size |
| Aldosary 2025 | Not reported. | Not reported. | Data available upon request from corresponding author | (1 )Datasets may not represent full range of PCOS manifestations. (2).Did not account for confounding variables (e.g., lifestyle, comorbidities). (3) PCOSNet performance dropped on Dataset III, indicating generalization challenges. (4).Need for improved model interpretability for clinical trust. |
| Narni 2025 | Not reported. | Not reported. | Not reported | Hospital-based study, which may limit the generalizability of findings to the broader population. |
| Panjwani 2025 | Not reported. | Not reported. | Data: Publicly available on Kaggle (links provided). Code: Not reported. | (1) Increased computational complexity and longer execution time of the proposed ensemble framework. (2) The need for a more exhaustive, high-quality dataset with a larger number of samples to test real-time robustness. |
| Rona 2025 | Partially yes. Radiologists performing ovary segmentation on MRI were blinded to the diagnosis. It is not explicitly stated if the clinicians applying the Rotterdam criteria (gold standard) were blinded to the radiomic model predictions. | Not reported. | Not reported | (1) Retrospective study design. (2) Relatively small patient cohort. (3) Manual segmentation of ovaries, which is time-consuming and can introduce interobserver variability. |
| Zad 2024 | Not reported. | Not reported. | Data is available upon reasonable request from corresponding author. | (1) Applicable only to individuals interacting with the healthcare system and deemed "at-risk," not for population-based screening; (2) Data sourced from a single urban hospital population, limiting generalizability to other settings (e.g., rural); (3)Potential for "informative presence" bias in EHR data, where data availability is linked to healthcare-seeking behavior and marginalization; (4)Possible need for stricter chart review beyond ICD-9 codes to clarify risk in some groups; (5) Exclusion of other potentially relevant predictors (diet, genetics, lifestyle);(6) Exclusion of concurrent endocrinopathies might be overly strict. (7)Did not evaluate Anti-Müllerian Hormone (AMH) as it was not widely used during the study period. |
| Agirsoy 2025 | Not reported. | Not reported. | Data: Publicly available on Kaggle (https://www.kaggle.com/datasets/prasoonkottarathil/polycystic-ovary-syndrome-pcos). Code: Not mentioned | (1) Prioritized a reduced, clinically meaningful feature set for applicability, which may result in marginally lower accuracy compared to studies using all features.(2) Used a real-world, heterogeneous dataset which may lead to lower performance metrics compared to studies using filtered/pre-balanced datasets.(3) Perfect scores on an external validation dataset raise concerns about potential overfitting or data leakage, necessitating further validation.(5)The study is retrospective; prospective validation is needed. (6) Generalizability to diverse ethnic groups, ultrasound devices, and clinical settings needs to be assessed. |
| Jaganathan 2025 | Not reported. | Not reported. | Data: Available (Public dataset from Kaggle). Code: Available (in Supplemental Files) | (1) Performance validated on a public dataset; needs confirmation with real-time data. (2)Sample size may be small, limiting generalizability to diverse populations. (3)Dataset may lack demographic diversity and suffer from data quality issues (missing/inaccurate recordings). (4)Legal, practical, and ethical obstacles for integrating the blockchain-based system into existing healthcare infrastructures. (5)Research focused on detection, not on treatment outcomes or long-term management. |
| Yao 2025 | Not reported. | Not reported. | Data: The original contributions presented in the study are included in the supplementary material. Further inquiries can be directed to the corresponding authors. Code: Not mentioned. | (1) Retrospective design with potential selection bias. (2) Moderate overall sample size; need for larger prospective validation. (3) Lack of anthropometric parameters (e.g., BMI, waist circumference). (4) Did not incorporate imaging or multi-omics data. (5) Limited generalizability as all participants were from China; needs validation in multi-ethnic cohorts. (6) Potential impact of menstrual cycle phase on hormonal measurements, though measurements were standardized to the follicular phase where possible. |
| Natarajan 2025 | Not reported. | Not reported. | Data: Publicly available on Kaggle. Code: Not explicitly stated | (1) Performance depends on high-quality ultrasound images. (2) Challenges with small/overlapping follicles and low contrast. (3) Limited to 2D images; does not leverage 3D/temporal data. (4) Potential misclassification in noisy or artifact-affected images |
| Sarwar 2025 | Not reported. | Not reported. | Data: Publicly available on Kaggle. Code: Available via Colab Notebook | (1) Use of quantum simulator (PennyLane) rather than real quantum hardware. (2) Computational and operational differences between simulation and real quantum systems. (3) Need for validation on emerging quantum computing platforms. (4) Potential overfitting concerns addressed by dataset split and regularization, but perfect accuracy may raise generalizability questions. |
| Bharti 2025 | Not reported. | Not reported. | Data publicly available (Kaggle links provided); code availability not mentioned. | (1) Increased computational complexity and longer execution time. (2) Dataset size limited (900 samples) for learning complex relationships. (3) Model performance may improve with larger, more diverse datasets. (4) Only symptomatic and routine clinical features used, not invasive hormonal/ultrasound data. |
| Ramteke 2025 | Not applicable (public dataset with pre-labeled outcomes) | Not reported. | Code Availability: Not available Data Availability: Publicly available on Kaggle | (1) Dataset lacks demographic diversity (e.g., ethnicity, socioeconomic status). (2) Single regional (likely Indian) dataset limits generalizability. (3) No external validation performed. (4) Potential overfitting concerns with high-dimensional features. (5) Model interpretability methods are model-specific and may not generalize |
| Sundari 2025 | Not reported. | Not reported. | Code Availability: Not available Data Availability: Publicly available (PCOSGen dataset via GitHub/MONAI) | (1) Relatively small and limited dataset. (2) Lack of external validation on multi-center, diverse populations. (3) Biomarker data not integrated into the final model (used only for visualization). (4) Potential overfitting despite regularization and augmentation. (5) Need for larger, more diverse datasets to improve generalizability. (6) Limited evaluation of model uncertainty and real-time clinical applicability. |
| Pulluraparbil 2026 | Not reported. | TRIPOD-AI checklist | Data: Publicly available on Kaggle, UCI, and other repositories Code: Not mentioned | (1) Dataset size and demographic diversity limited. (2) No prospective validation. (3) Lack of multi-center external validation. (4) Computationally intensive pipeline (GS2TM + DASCD). (5) No integration of raw ultrasound imaging data. (6) Feature redundancy may still exist despite selection. |
| Uddin 2026 | Not reported. | Not reported. | Code and data are publicly available. GitHub repository link is provided in the "Code and data availability" section. | (1) Limited dataset diversity (geographically concentrated, lack of multi-ethnic representation). (2) Reliance on SMOTE-generated synthetic data, which may not fully capture real-world complexity. (3)The web-based interface requires internet access and digital literacy, potentially limiting access in low-resource settings. (4) Explainability tools (SHAP/LIME) may be difficult for clinicians without AI expertise to interpret. |
