# Supplementary material for: Machine learning and deep learning for diagnosis of Polyendocrine Metabolic Ovarian Syndrome: systematic review and meta-analysis
Source: Front Endocrinol (Lausanne). 2026 Jul 30;17:1889375. doi: 10.3389/fendo.2026.1889375 (PMC13468022; doi:10.3389/fendo.2026.1889375)
Supplement: Supplementary file 7 [file SupplementaryFile5.docx]

**Supplementary Data 5. The characteristic of patients in included studies**

| **Study** | **Total number of cases** | **Baseline characteristics (case group)** | **Definition of the control group** | **Characteristics (control group)** | **Data collection period** |
| --- | --- | --- | --- | --- | --- |
| Cheng 2019 | 39,093 ultrasound reports | Age (mean ± SD): 31.4 ± 7.9 years (entire cohort). BMI (n=18,443): 27.9% normal, 30.4% overweight, 40.5%. | "PCOM-absent" based on the Rotterdam criteria applied to the ultrasound report text. | Baseline characteristics are provided for the entire source population (Table 1), not stratified by PCOM status | October 1, 2003 to December 7, 2016 |
| Lv 2022 | 721 women | Not reported. | Subjects without a PCOS diagnosis | Not reported | 2017 to 2019 |
| Zhang 2021 | 100 women | Age (years): 30.02 ± 3.47; BMI (kg/m²): 26.47 ± 3.76;Total Testosterone (ng/ml): 0.75 ± 0.24; LH (IU/l): 10.42 ± 4.79; Fasting Insulin (mU/l): 16.02 ± 8.15; LH/FSH Ratio: Implied from LH (10.42) and FSH (7.11); Triglycerides (mmol/l): 1.90 ± 1.44. | Healthy (non-PCOS) women undergoing IVF for tubal or male factor infertility, with regular menstrual cycles and normal ovarian reserve. Other endocrine disorders were excluded. | Age: 30.24 ± 3.24; BMI: 22.33 ± 3.70; Total Testosterone: 0.47 ± 0.14; LH: 5.66 ± 3.01; Fasting Insulin: 10.69 ± 4.87. | Not reported |
| Xie 2022 | 60 women | Subjects were age-matched. | Age-matched healthy women. | Not reported | Not reported |
| Sreejith 2022 | 541 women | Only a list of features is provided in Table 1, without mean/proportion values for cases vs. controls | Not specified. | Not reported | Not reported |
| Xu 2022 | 11,720 cycles | Age: 31.4 ± 5.0 years; BMI: 24.1 (IQR 21.5–26.8) kg/m²; Menstrual cycle upper limit (UML): 40 (IQR 30–62.5) days; AMH: 6.4 (IQR 3.5–10.1) ng/mL; Androstenedione (AND): 8.9 ± 4.9 nmol/L; Testosterone (TES): 0.7 (IQR 0.7–1.1) nmol/L; AFC: 10 (IQR 6–14). | Infertility patients without PCOS | Not reported | January to December 2019 |
| Suha 2022 | 594 ovary ultrasound images | Not reported. | Non-PCOS ultrasound images (labeled by specialists). | Not reported | Not reported |
| Mehr 2022 | 541 women | Not reported. | Non-PCOS patients from the same dataset. | Not reported | Not reported |
| Zigarelli 2022 | 526 women | Age: median 29 years (IQR 26.25–33); BMI: median 25.1 kg/m² (IQR 26.25–33); Irregular menstrual cycle: 53.5%; Hirsutism: 12.9%; Acne: 69.4%; Weight gain: 68.8%; Acanthosis nigricans: 61.8%; AMH: median 1.74 ng/mL (IQR 0.88–2.32). | Women without PCOS diagnosis from same hospitals. | Not reported | Not reported |
| Silva 2021 | 145 women | Age: 30.6 ± 5.2 years; BMI: 28.4 ± 8.3 kg/m²; Abdominal Circumference: 96.5 (15.4) cm (median, IQR); Testosterone: 53.30 (48.08) nmol/L (median, IQR); Dyslipidemia: 9.7% (proportion). | Healthy women with regular menstrual cycles, no clinical or biochemical hyperandrogenism, and no evidence of polycystic ovaries on ultrasound. | Age: 28.8 ± 7.2 years; BMI: 23.1 ± 5.3 kg/m²; lower testosterone and dyslipidemia prevalence (0%) compared to PCOS group. | 2011 to 2013 |
| Tiwari 2022 | 541 women | Age, weight, BMI, cycle length, follicle count, hormone levels (LH, FSH, AMH, etc.), endometrial thickness, etc.. | Not specified. | Not reported | Not reported |
| Alamoudi 2023 | Ultrasound images: 391 (127 PCOM, 264 normal) Clinical data: 285 patients (129 PCOS, 156 non-PCOS) | Demographic: Age, marital status Vital signs: BMI, weight, height Laboratory: FSH, Testosterone Clinical: Cycle regularity | Normal ovaries or non-PCOS based on radiologist assessment and clinical diagnosis. | Not reported | Not reported |
| Wang 2023 | 486 women (285 PCOS patients, 201 non-PCOS) | Age: 31.000 (6.000) years Height: 1.620 (0.030) m Weight: 62.100 (8.800) kg BMI: 24.221 (2.384) kg/m² | Healthy female volunteers from the Physical Examination Center of the same hospital, without PCOS or other significant diseases. | Age: 28.000 (5.000) years; Height: 1.640 (0.080) m; Weight: 61.000 (9.200) kg; BMI: 22.684 (2.534) kg/m². | Not reported |
| Nsugbe 2023 | Original dataset: 541 records (177 PCOS, 364 non-PCOS). After SMOTE balancing: 728 samples | Not reported. | Defined as patients without PCOS from the Kaggle dataset. | Not reported | Not reported |
| Suha 2023 | Original dataset: 541 records (177 PCOS, 364 non-PCOS). After SMOTE balancing: 728 samples (364 each class) | Not reported (only age range 20-50 years is mentioned in data visualization). | Defined as "non-PCOS" patients from the Kaggle dataset. | Not reported | Not reported. |
| Lim 2023 | 459 women (316 PCOS, 143 non-PCOS) | Age: 27.00 (24.00, 29.75) years; BMI: 22.03 (19.91, 25.08) kg/m². | Healthy females with none of the PCOS diagnostic criteria and free from gynecological/organic diseases. | Baseline (median [P25, P75]): Age: 25.00 (21.00, 31.00) years; BMI: 20.44 (19.15, 21.77) kg/m². | August 2018 to January 2022 |
| Lim 2023 | 450 women (294 PCOS, 156 non-PCOS) | Age: 28.0 (25.0, 31.0) years; Weight: 58.0 (52.0, 65.0) kg; BMI: 22.1 (19.9, 24.8) kg/m². Menstrual irregularities: Oligomenorrhea 62.2%, Hypomenorrhea 9.9%, Combined 27.9%. | Non-PCOS females with irregular menstruation (oligo-, hypo-, or combined). | Baseline (Median [P25, P75]): Age: 30.0 (27.0, 33.0) years; Weight: 54.0 (49.0, 60.0) kg; BMI: 20.7 (18.8, 22.6) kg/m². Menstrual irregularities: Oligomenorrhea 39.7%, Hypomenorrhea 50.6%, Combined 9.6%. | August 2018 to January 2022. |
| Sheikdavood 2023 | 541 women | Age, BMI,Menstrual irregularity, Hirsutism, Biochemical markers: Includes AMH, LH, FSH, etc. | Healthy group defined based on clinical and metabolic attributes without PCOS. | Not reported | Not reported |
| Elmannai 2023 | 541 women | Age, Weight, Height, BMI, Cycle length, Follicle number (left/right), Hormonal levels (FSH, LH, AMH, TSH, PRL, etc.), Clinical symptoms (hair growth, skin darkening, weight gain, pimples, hair loss), Lifestyle (fast food, regular exercise). | Defined as not having PCOS based on the dataset labeling. | Not reported | Not reported |
| Kumari 2023 | 541 women | Age, Weight, BMI, Cycle regularity, Hormonal levels (FSH, LH, TSH, AMH, PRL, etc.), Clinical symptoms (hair growth, skin darkening, weight gain, pimples, etc.). | Defined as not having PCOS based on dataset labeling. | Not reported | Not reported |
| Kumar 2024 | 541 women | Key attributes are listed (e.g., age, BMI, cycle regularity, hormonal levels, symptoms like hair growth). However, mean or proportion values for baseline characteristics (age, BMI, etc.) are not explicitly reported in the provided text. The analysis indicates the average age of PCOS patients was ~30 years. | Not specified. | Not reported | Not reported |
| Nethra 2024 | Ultrasound images: 3,200 for training, 1,468 for testing Clinical data: 1,468 patients in final prediction | Age, Weight, Height, BMI, Cycle length, Weight gain, Skin darkening, Hair growth, Hair loss, Pimples, Fast food consumption (specific values not provided). | Not specified. | Not reported | Not reported |
| Moral 2024 | Kaggle dataset: 1,924 images (781 PCOS,143 non-PCOS) PCOSGen dataset: 3,200 non-PCOS,1,468 PCOS MMOTU dataset: 1,639 images from 294 patients | Not reported. | Healthy ovaries labeled as “NOT INFECTED” in Kaggle dataset. | Not reported | Not reported |
| Kermanshahchi 2024 | 1,932 pelvic ultrasound images (PCOS-positive: 787 images, Normal: 1,145 images) | Not reported. | Normal pelvic ultrasound images. | Not reported | Not reported |
| Li 2024 | 28 (15 PCOS, 13 non-PCOS) | Combined samples from GSE137684 and GSE155489 (exact number not specified in the provided text). | Defined as "normal samples" from the GEO datasets. | Not reported | Not reported |
| Rahman 2024 | 541 (177 PCOS, 364 non-PCOS) | Not reported in detail (only mutual information scores for features provided). | Defined as PCOS-negative based on dataset labeling (“0” for negative, “1” for positive). | Not reported | Not reported |
| Sowmiya 2024. | Dataset1: 100 (50 PCOS, 50 non-PCOS) Dataset2: 200 (100 PCOS, 100 non-PCOS) | Age range: 18–45 years Clinical features: multiple ovarian follicles, irregular periods. | Healthy volunteers without PCOS (confirmed by ultrasound). | Not reported | March to April 2024 (Dataset2); Dataset1 timeframe not specified |
| Rona 2024 | Total Sample Size: 101 PCOS patients (202 ovaries) : Case Group (Classical PCOS): 65 patients (130 ovaries) Control Group (Non-classical PCOS): 36 patients (72 ovaries) | Age: 23.18 ± 4.42 years BMI: 28.17 ± 6.82 Oligo-amenorrhea: 98.46% Hyperandrogenism: 70.76% FG score (median): 10 Testosterone (median): 64 ng/dl | Non-classical PCOS (phenotypes C & D) based on NIH phenotype classification. | Age: 22.36 ± 4.13 years; BMI: 26.29 ± 5.82; Oligo-amenorrhea: 30.55%; Hyperandrogenism: 94.44% FG score (median): 8; Testosterone (median): 53.50 ng/dl. | January 2014 to June 2022 |
| Paramasivam 2024 | 277 images (149 PCOS, 128 non-PCOS) | Not reported. | Non-PCOS ultrasound images (presumably normal ovaries). | Not reported | Not reported |
| Shamnugavadivel 2024 | Total Sample Size (Clinical): 541 patients (177 PCOS, 364 non-PCOS) Total Sample Size (Ultrasound Images): 3856 images (1568 PCOS, 2288 C) | Not reported (only features listed, no summary statistics). | Patients/images labeled as non-PCOS in the Kaggle dataset. | Not reported | Not reported |
| Wang 2024 | 130 patients (70 PCOS, 60 non-PCOS ) | (median/mean):Age: 26.786 ± 4.259 years;BMI: 22.547 kg/m²;LH: 13.030 mIU/L;LH/FSH: 2.446;AMH: 6.940 ng/mL;HOMA-IR: 2.316;HDL-C: 1.270 mmol/L. | Age-matched healthy women without PCOS. | Age 27.783 ± 5.970 years, BMI 21.484 kg/m², normal hormone/metabolic profiles. | April 2022 to June 2023 |
| Rajput 2024 | 541 (177 with PCOS, 364 Non-PCOS ) | Age range: 20-48 years (no mean provided);nBMI range: 15.1–64.28 (no mean); Other clinical features: menstrual irregularity, hirsutism, acne, etc. (binary categorical variables); Hormonal levels: FSH, LH, FSH/LH, PRL, PRG, TSH, Vit D3 included in dataset. | Non-PCOS individuals from same dataset. | Not reported | Not reported |
| Vairachilai 2024 | 541 women (177 with PCOS, 364 non-PCOS). | Age range: Provided in dataset but mean/median not specified in text; Specific values for BMI, menstrual irregularity, hirsutism, and biochemical markers are not provided in the results section. The dataset includes these features. | Women from the same dataset without a PCOS label. | Not reported | Not reported |
| Umaa 2024 | 541 (364 non-PCOS, 177 PCOS), after data balancing (SMOTE): 724 records (362 PCOS, 362 non-PCOS) | Age: most common 28 years; BMI: varied, most common 23.11 Menstrual Irregularity (Cycle R/I): 149 irregular (1), 389 regular (0); Hirsutism (Hair growth Y/N): 148 yes, 390 no; Biochemical: FSH, LH, AMH, TSH, PRL, Hb, RBS, etc.. | Patients without PCOS diagnosis (‘No’ in PCOS(Y/N) column). | Similar demographic and clinical parameters as cases. | Not reported |
| Ahmad 2024 | 541women | Not explicitly reported in detail; dataset includes features such as Age, BMI, menstrual irregularity, hirsutism, hormonal levels (FSH, LH, AMH, etc.), and ultrasound measures (follicle count, size). | Patients labeled as "0" (non-PCOS) in the dataset. | Similar feature set as cases; no detailed statistics provided. | Not reported |
| Sumathi 2023 | 1918 ultrasound images (781 PCOS, 1137 normal) | Not reported. | Normal ultrasound images (no PCOS). | Not described (image-based only). | Not reported |
| Tong 2025 | 159 participants (84 PCOS cases, 75 healthy controls) | Age: 29.00 ± 3.75 years; BMI: 22.95 (20.5, 26.2) kg/m² [median (P25, P75)]; LH: 10.45 (7.22, 14.94) IU/L; LH/FSH: 2.02 (1.35, 2.81); T: 2.44 (1.94, 2.86) nmol/L; AMH: 9.93 (6.79, 12.96) ng/mL. | Healthy individuals from the same hospital's Health Screening Centre, matched for age and BMI, with no history of hormonal drug use, endocrine diseases, etc.. | Baseline (n=65 for modeling): Age: 30.05 ± 3.83 years; BMI: 22.32 (20.10, 25.20) kg/m². | May 2023 to November 2023 |
| Smithakranthi2025 | 541women | Specific mean/median values for age, BMI, menstrual disorder/hirsutism proportions, and biochemical markers like LH, FSH, Testosterone are not provided in the extracted tables/sections of this manuscript. | Fertile women from the same Kaggle dataset who do not have PCOS. | Specific baseline characteristics for the control group are not provided in the extracted tex | Not reported |
| Oviya 2025 | 541women | Not reported. | Non-PCOS individuals from the same Kaggle dataset. | Not reported | Not reported |
| Ghosh 2025 | 3846 images (2284 Non-PCOS, 1562 PCOS) | Menstrual irregularity, hirsutism, biochemical markers: Not detailed. | Non-PCOS images from the same Kaggle dataset | Not reported | Not reported |
| Faris 2025 | 541 women (177 with PCOS, 364 non-PCOS) | Age: 20–48 years (range)  BMI: Not reported as mean/proportion  Menstrual irregularity, hirsutism, biochemical markers: not detailed numerically | Non-PCOS women from the same dataset (healthy subjects). | Not reported | Not reported |
| Aldosary 2025 | Dataset I: 541 women (177 PCOS, 364 non-PCOS) Dataset II: 23 samples (10 PCOS, 13 non-PCOS) Dataset III: 138 samples (augmented from 23 original; 828 after Mixup, 1656 after SMOTE) | Not reported in detail (only features listed, e.g., Age, BMI, Cycle length, AMH, Follicle No., etc.). No means/proportions provided for PCOS group specifically. | Non-PCOS women (Dataset I), Healthy controls (Datasets II & III). | Not reported | Not reported |
| Narni 2025 | 200 (100 PCOS, 100 non-PCOS) | Age: Mean 26.51 (SD 2.50); Other features (Proportion): All features were listed as odds ratios. Specific proportions for the case group (e.g., % with hirsutism, irregular cycles) are not reported numerically. | Women without a PCOS diagnosis based on ultrasonography. | Age: Mean 26.76 (SD 2.48). Authors state groups were age-matched. | Not reported |
| Panjwani 2025 | 900 samples (after outlier removal from initial 932) | Not reported. | Not specified. | Not reported | Not reported |
| Rona 2025 | 101 PCOS patients (202 ovaries), 40 healthy controls (78 ovaries) | Age: Mean 23 ± 4 years; BMI: Mean 28 ± 12 kg/m²; Oligo-amenorrhea: 76.9%; Clinical Hyperandrogenism (FG score ≥7): 75.8%; Biochemical Hyperandrogenism: 62.9%; Median Testosterone: 60 ng/dl (IQR 45-75); PCOM on US: 85.2%. | Age- and BMI-matched healthy women without clinical/biochemical hyperandrogenism, with regular menstrual cycles, examined for euthyroid multinodular goiter. | Age: Mean 24 ± 5 years; BMI: Mean 29.06 ± 16 kg/m²; Oligo-amenorrhea: 1.1%; Clinical Hyperandrogenism (FG score ≥7): 4.3%; Biochemical Hyperandrogenism: 9.9%; Median Testosterone: 38 ng/dl (IQR 27-47); PCOM on US: 24.1%. | January 2014 to June 2022 |
| Zad 2024 | 30,601 women | Age (Mean ± SD): 31.3 ± 6.6 years; Race/Ethnicity: Black/African American (40.5%), White (26.4%), Hispanic/Latina (9.6%), Asian (4.6%); BMI Categories: Normal (<25 kg/m²): 25.8%, Overweight (25-30 kg/m²): 19.4%, Obese (≥30 kg/m²): 26.1%, Unknown: 28.7%; Prevalence of Diagnostic Criteria : Model I (ICD-9 code): 4.5% (1,329); Model II (IM+HA, no code): 3.6% (1,056); Model III (Rotterdam+, no code): 3.8% (1,116) | Did not have the ICD-9 code and did not meet the specified Rotterdam criteria. | Not reported | October 2003 to December 2016 |
| Agirsoy 2025 | 541 women | Significantly higher weight, BMI, cycle length, hair growth (hirsutism), skin darkening, weight gain, and follicle count in both ovaries compared to non-PCOS group. AMH levels were a top predictor; Features like hair growth, skin darkening, weight gain, and menstrual irregularity are highlighted as strong differentiating factors. | Defined as "non-PCOS" cases within the same dataset of women evaluated for suspected PCOS. They are the counterpart to the PCOS group. | Not reported | Not reported |
| Jaganathan 2025 | 541 women | Not reported. | Not specified. | Not reported | Not reported |
| Yao 2025 | 1,883 women (Training cohort: 1,600; External validation cohort: 283) | Age (years) (Median, Q1, Q3): 28 (25, 32); Biochemical Indicators (Median, Q1, Q3): AMH (ng/ml): 5.64 (2.87, 9.7); DHEAS (μg/dL): 189.12 (136.44, 268.74);T (nmol/L): 1.05 (0.73, 1.44); LH (IU/L): 5.3 (3.09, 9.48); FSH (IU/L): 5.14 (4.12, 6.35); TG (mmol/L): 1.04 (0.73, 1.53) | Non-PCOS women from the same clinics who did not meet the Rotterdam criteria. | Distributions of age and biochemical markers were similar to the PCOS group (no statistically significant differences for most variables) | Training Cohort: January 2021 to January 2025; External Validation Cohort: January 2024 to January 2025 |
| Natarajan 2025 | 3,800 ultrasound images (1,900 PCOS, 1,900 non-PCOS). | Not reported. | Healthy ultrasound images from the same dataset, labeled as “Normal healthy images.” | Not reported | Not reported |
| Sarwar 2025 | 3,846 ultrasound images (1,562 PCOS, 2,284 non-PCOS) | Not reported. | “Not infected” ultrasound images from the same dataset, labeled as normal ovarian tissue | Not reported | Not reported |
| Bharti 2025 | 900 samples | Waist-to-hip ratio, weight gain, BMI, cholesterol, hair growth, cycle length, etc. Specific means/proportions not reported. | Patients without PCOS. | Not reported | Not reported |
| Ramteke 2025 | 541 samples (177 PCOS, 364 non-PCOS) | Not reported. | Labeled as "No" in the dataset (non-PCOS). | Not reported | Not reported |
| Sundari 2025 | 3200 | Not reported. | Normal ovarian morphology on ultrasound. | Not reported | Not reported |
| Pulluraparbil 2026 | Kaggle dataset: 541 (177 PCOS, 364 non-PCOS) UCI PCOS dataset: 541 | Not reported in detail; selected features include age, BMI, follicle count, hormonal levels (FSH, LH, AMH, PRL, etc.), menstrual regularity, hair growth, weight gain, etc. | Non-PCOS cases from the same datasets. | Not reported | Not reported |
| Uddin 2026 | 541 (177 PCOS, 364 non-PCOS) | Not reported. | Defined as non-PCOS patients within the same dataset. | Not reported | Not reported |
