# Supplementary material for: Machine learning and deep learning for diagnosis of Polyendocrine Metabolic Ovarian Syndrome: systematic review and meta-analysis
Source: Front Endocrinol (Lausanne). 2026 Jul 30;17:1889375. doi: 10.3389/fendo.2026.1889375 (PMC13468022; doi:10.3389/fendo.2026.1889375)
Supplement: Supplementary file 8 [file SupplementaryFile6.docx]

**Supplementary Figure 1**. Methodological assessment by Quality Assessment of Diagnostic Accuracy Studies-2 (QUADAS-2). Summary of the risk of bias for each study. Green, yellow, and red circles indicate low, unclear, and high risk of bias, respectively
