# Supplementary material for: Machine learning and deep learning for diagnosis of Polyendocrine Metabolic Ovarian Syndrome: systematic review and meta-analysis
Source: Front Endocrinol (Lausanne). 2026 Jul 30;17:1889375. doi: 10.3389/fendo.2026.1889375 (PMC13468022; doi:10.3389/fendo.2026.1889375)
Supplement: Supplementary file 10 [file SupplementaryFile8.docx]

**Supplementary Figure 3. Forest plot of pooled specificity for machine learning (ML) and deep learning (DL) models in diagnosing Polyendocrine Metabolic Ovarian Syndrome (PMOS).** This analysis includes 60 datasets from 56 studies published between 2019 and 2026, with study populations originating from 14 countries. The pooled specificity was 0.94 (95% CI: 0.91-0.96), estimated using a random-effects model with the Hartung-Knapp-Sidik-Jonkman (HKSJ) method. Substantial heterogeneity was observed (I² = 93.4%). The 95% prediction interval ranged from 53% to 100%.
