# Supplementary material for: Machine learning and deep learning for diagnosis of Polyendocrine Metabolic Ovarian Syndrome: systematic review and meta-analysis
Source: Front Endocrinol (Lausanne). 2026 Jul 30;17:1889375. doi: 10.3389/fendo.2026.1889375 (PMC13468022; doi:10.3389/fendo.2026.1889375)
Supplement: Supplementary file 11 [file SupplementaryFile9.docx]

**Supplementary Figure 4. Forest plot of pooled diagnostic odds ratio (DOR) for machine learning (ML) and deep learning (DL) models in diagnosing Polyendocrine Metabolic Ovarian Syndrome (PMOS).** Based on 60 datasets from 56 studies published between 2019 and 2026, with study populations originating from 14 countries, the pooled DOR was 202.13 (95% CI: 100.97-404.67), estimated using a random-effects model with the Hartung-Knapp-Sidik-Jonkman (HKSJ) method. High heterogeneity was observed (I² = 93.6%).
